# 23-valent polysaccharide vaccine (PPSV23)-targeted serotype-specific identification of *Streptococcus pneumoniae* using the loop-mediated isothermal amplification (LAMP) method

**DOI:** 10.1371/journal.pone.0246699

**Published:** 2021-02-16

**Authors:** Jiwon Lee, Youngbae Yoon, Eun Jin Kim, Donghyun Lee, Yeongjun Baek, Chika Takano, Bin Chang, Takahiro Iijima, Paul E. Kilgore, Satoshi Hayakawa, Tomonori Hoshino, Dong Wook Kim, Mitsuko Seki

**Affiliations:** 1 Division of Pediatric Dentistry, Department of Human Development and Fostering, Meikai University School of Dentistry, Saitama, Japan; 2 Department of Pharmacy, College of Pharmacy, Hanyang University, Ansan, Republic of Korea; 3 Institute of Pharmacological Research, Hanyang University, Ansan, Republic of Korea; 4 Division of Microbiology, Department of Pathology and Microbiology, Nihon University School of Medicine, Tokyo, Japan; 5 Bacteriology I, National Institute of Infectious Diseases, Tokyo, Japan; 6 Department of Pharmacy Practice, Eugene Applebaum College of Pharmacy & Health Sciences, Wayne State University, Detroit, MI, United States of America; Defense Threat Reduction Agency, UNITED STATES

## Abstract

Reports of invasive disease due to *Streptococcus pneumoniae* have declined since the introduction of pneumococcal conjugate vaccines (PCV7 and PCV13). The incidence of invasive diseases due to *S*. *pneumoniae* that are not addressed by the vaccines, however, has increased in children and adults, creating a global public health problem. Previously, we established the loop-mediated isothermal amplification (LAMP) method for a PCV13 serotype-specific assay. In the current study, we developed a rapid, simple, and cost-effective assay to detect serotypes in the 23-valent pneumococcal polysaccharide vaccine (PPSV23) using the LAMP method. In this study, LAMP primer sets for serotypes 2, 8, 9N, 10A, 11A, 12F, 15B, 17F, 20, 22F, and 33F of *S*. *pneumoniae* were developed. The reactivity, specificity, and sensitivity of LAMP assays were determined and compared to those of conventional PCR. The feasibility of LAMP assays in clinical application in patients with invasive pneumococcal diseases was validated by defining the detection limit of the LAMP assay with bacterial genomic DNA-spiked blood specimens. The specificity of each LAMP assay was determined using 44 serotypes of pneumococcal strains. Their sensitivity was 100 copies per reaction versus 10^3^ to 10^6^ copies per reaction for PCR assays. Using DNA-spiked blood specimens, excluding the LAMP assay that targeted serotype 22F (10^3^ copies per reaction), the limit of detection of the LAMP assay was similar to that with purified DNA as the template (10^2^ copies per reaction), compared with 10^3^ to >10^6^ copies per reaction for PCR assays. In conclusion, a rapid and simple LAMP-based PPSV23-targeted serotype detection assay was developed for use in many countries. This study is the first report of a LAMP-based assay for identification of PPSV23 serotypes. Further evaluation of this assay is needed through surveillance and vaccine efficacy studies.

## Introduction

Pneumonia is an important infectious disease with high morbidity and mortality [1, 2]. *Streptococcus pneumoniae* can lead to serious invasive diseases, such as meningitis, septicemia, and pneumonia, as well as milder but more common illnesses, such as otitis media [3]. *S*. *pneumoniae* frequently colonizes the human nasopharynx, and infants and young children are the main reservoir of this organism. Higher carriage rates among children in low- and middle-income countries and immunocompromised adults in high-income countries have been reported [4, 5]. A 23-valent polysaccharide vaccine (PPSV23) was introduced in the early 1980s, and pneumococcal conjugate vaccines (PCV7 or 13) have been available for children younger than 5 years since 2009; at least 10 other PCVs, containing 10–20 serotypes, are undergoing clinical trials [6]. PPSV23 contains 12 of the serotypes in PCV13 and an additional 11 serotypes (2, 8, 9N, 10A, 11A, 12F, 15B, 17F, 20, 22F, and 33F). PPSV23 is recommended for prevention of invasive pneumococcal diseases (IPDs) among all adults aged ≥ 65 years and in high-risk adults aged 19–64 years [5]. Administration of PCV to those older than 5 years may be useful in control of outbreaks in older children and adults [7].

Since the introduction of PCVs, IPDs in children have decreased [8, 9]. However, non-PCV serotype strains have become an emerging problem in high-income countries [9], and IPD-associated mortality in adults and children remains significant in low- and middle-income countries [10].

The capsular polysaccharide is an essential virulence factor of *S*. *pneumoniae*, and pneumococcal serotypes are defined based on differences in structure. There are approximately 94 serotypes, and their dissemination varies geographically [11, 12]. Current PCVs have been developed against the most prevalent invasive serotypes identified in surveillance studies [12]. However, after introductions of PCVs, IPDs caused by non-PCV types increased [13]. In addition, regarding the impact and cost-effectiveness of PPSV23, there are various opinions and no consensus on use of this vaccine [14]. The WHO recommends that the epidemiological impact of pneumococcal vaccines be carefully monitored by sustained, high-quality sentinel and population-based surveillance of pneumococcal disease [6].

Conventional bacterial culture of *S*. *pneumoniae* requires specialized bacterial culture media and reagents and should be performed according to the laboratory biosafety manual [15]. The Quellung reaction using type-specific pneumococcal antisera has been routinely used in serological typing of pneumococci. Swelling of the bacterial capsule by type-specific antisera is scored under a microscope in this conventional determination. However, the method cannot be used on culture-negative samples. Additionally, the precise reactions require advanced technical skill, and errors can occur without strict control of antisera. Accurate determination of pneumococcal serotyping remains problematic in low- and middle-income countries with limited routine microbiology laboratory services and facilities [16]. Therefore, effective serotype identification tests of *S*. *pneumoniae* that could be routinely utilized are desired.

Molecular methods can be a useful alternative, and multiplex polymerase chain reaction (PCR)-based assays for identification of serotypes of *S*. *pneumoniae* have been developed and successfully identified pneumococcal serotypes when standard culture methods failed [17]. Loop-mediated isothermal amplification (LAMP) is a nucleic acid amplification method useful in diagnosis of various infectious diseases and is one of the most specific, sensitive, robust, and easy to use molecular methods [18, 19]. Pneumococcal serotyping could be further improved using LAMP.

Diagnosis of *S*. *pneumoniae*, *Haemophilus influenzae*, and *Neisseria meningitidis* in cerebrospinal fluid (CSF) using LAMP assay has been reported previously [20–23]. Furthermore, LAMP assays for discrimination of meningococcal serogroups (A, B, C, X, Y, and W) and *H*. *influenzae* serotypes (a, b, c, d, e, and f) have been established [20, 24, 25]. Finally, LAMP methods to detect capsular types 1, 3, 4, 5, 6A, 6B, 7F, 9V, 14, 18C, 19A, 19F, and 23F (PCV7 or PCV13 vaccine-targeted serotypes) have been developed [26]. However, there are no available LAMP methods to serotype PPSV23-targeted pneumococcus. In this study, to understand the epidemiological impact of PPSV23, we established LAMP primer sets targeting the same genes as in conventional multiplex PCR-based assays to detect capsular types 2, 8, 9N, 10A, 11A, 12F, 15B, 17F, 20, 22F, and 33F of *S*. *pneumoniae* [27–29].

## Materials and methods

### Bacterial strains

Fifty-five strains of *S*. *pneumoniae*, including serotypes that belong to PCV7, PCV13, and PPSV23 and non-vaccine serotypes, were analyzed in this study (Table 1). The PCV7 serotypes were 4 (SP0143), 6B (SP1489), 9V (SP2928), 14 (SP3320), 18C (SP2818), 19F (SP1118), and 23F (SP2838). Six additional serotypes in PCV13 were 1 (SP3121), 3 (SP1441), 5 (ATCC 6305), 6A (SP1567), 7F (SP3365), and 19A (SP1516). Eleven serotypes that belonged to PPSV23 in addition to PCV13 were 2 (D39, P01-10), 8 (ATCC 6308, ASP1012), 9N (SP2700, SP1926), 10A (SP1933, SP2000), 11A/E (SP2760, SP3845), 12F (SP0113, SP3716), 15B (SP3354, SP3850), 17F (NCTC 11904, SP3846), 20 (SP2830, SP3847), 22F (SP1854, SP3777), and 33F (SP3201, SP3766). The non-vaccine serotypes were 6C (SP3362), 6D (SP2739), 7C (SP3285), 9L (T9233/128/68), 11D (SP0510), 12A (559/66), 13 (SP0073), 15A (SP2758), 15C (SP3343), 18B (SP1901), 22A (3405/39), 23A (SP3374), 24F (SP3193), 33A (Biehl), 34 (SP3359), 35B (SP3357), 37 (SP2742), 38 (SP3356), 44 (Hammer), and 46 (Eddy nr. 73). Two non-pneumococcal species, *Haemophilus influenzae* (IID984) and *Neisseria meningitidis* (HY0001), also were analyzed.

**Table 1:** Reactivity and specificity of the pneumococcal serotyping LAMP assay.

Vaccine	Serotype	No. of strains	Strain ID	Origin of isolate	LAMP primer set										
					2	8	9N	10A	11A	12F	15B	17F	20	22F	33F
PCV7	4	1	SP0143	CSF	-	-	-	-	-	-	-	-	-	-	-
	6B	1	SP1489	N	-	-	-	-	-	-	-	-	-	-	-
	9V	1	SP2928	B	-	-	-	-	-	-	-	-	-	-	-
	14	1	SP3320	B	-	-	-	-	-	-	-	-	-	-	-
	18C	1	SP2818	B	-	-	-	-	-	-	-	-	-	-	-
	19F	1	SP1118	N	-	-	-	-	-	-	-	-	-	-	-
	23F	1	SP2838	N	-	-	-	-	-	-	-	-	-	-	-
PCV13	1	1	SP3121	B	-	-	-	-	-	-	-	-	-	-	-
	3	1	SP1441	N	-	-	-	-	-	-	-	-	-	-	-
	5	1	ATCC 6305	U	-	-	-	-	-	-	-	-	-	-	-
	6A	1	SP1567	N	-	-	-	-	-	-	-	-	-	-	-
	7F	1	SP3365	B	-	-	-	-	-	-	-	-	-	-	-
	19A	1	SP1516	N	-	-	-	-	-	-	-	-	-	-	-
PPSV23	2	2	D39, P01-10	U, U	+	-	-	-	-	-	-	-	-	-	-
	8	2	ATCC 6308, ASP1012	U, B	-	+	-	-	-	-	-	-	-	-	-
	9N	2	SP2700, SP1926	CSF, B	-	-	+	-	-	-	-	-	-	-	-
	10A	2	SP1933, SP2000	N, N	-	-	-	+	-	-	-	-	-	-	-
	11A	2	SP2760, SP3845	S, RT	-	-	-	-	+	-	-	-	-	-	-
	12F	2	KSP0113, SP3716	B, RT	-	-	-	-	-	+	-	-	-	-	-
	15B	2	SP3354, SP3850	RT, RT	-	-	-	-	-	-	+	-	-	-	-
	17F	2	NCTC 11904, SP3846	U, RT	-	-	-	-	-	-	-	+	-	-	-
	20	2	SP2830, SP3847	RT, RT	-	-	-	-	-	-	-	-	+	-	-
	22F	2	SP1854, SP3777	RT, RT	-	-	-	-	-	-	-	-	-	+	-
	33F	2	SP3201, SP3766	RT, RT	-	-	-	-	-	-	-	-	-	-	+
Non-vaccine serotype	6C	1	SP3362	N	-	-	-	-	-	-	-	-	-	-	-
	6D	1	SP2739	N	-	-	-	-	-	-	-	-	-	-	-
	7C	1	SP3285	N	-	-	-	-	-	-	-	-	-	-	-
	9L	1	T9233/128/68	U	-	-	+	-	-	-	-	-	-	-	-
	11D	1	SP510	RT	-	-	-	-	+	-	-	-	-	-	-
	12A	1	559/66	U	-	-	-	-	-	+	-	-	-	-	-
	13	1	SP0073	N	-	-	-	-	-	-	-	-	-	-	-
	15A	1	SP2758	RT	-	-	-	-	-	-	-	-	-	-	-
	15C	1	SP3343	RT	-	-	-	-	-	-	+	-	-	-	-
	18B	1	SP1901	N	-	-	-	-	-	-	-	-	-	-	-
	22A	1	3405/39	U	-	-	-	-	-	-	-	-	-	+	-
	23A	1	SP3374	N	-	-	-	-	-	-	-	-	-	-	-
	24F	1	SP3193	N	-	-	-	-	-	-	-	-	-	-	-
	33A	1	Biehl	U	-	-	-	-	-	-	-	-	-	-	+
	34	1	SP3359	N	-	-	-	-	-	-	-	-	-	-	-
	35B	1	SP3357	N	-	-	-	-	-	-	-	-	-	-	-
	37	1	SP2742	N	-	-	-	-	-	-	-	-	-	-	+
	38	1	SP3356	N	-	-	-	-	-	-	-	-	-	-	-
	44	1	Hammer	U	-	-	-	-	-	+	-	-	-	-	-
	46	1	Eddy nr. 73	U	-	-	-	-	-	+	-	-	-	-	-
Non-pneumococcal species															
*Haemophilus influenzae* IID984					-	-	-	-	-	-	-	-	-	-	-
*Neisseria meningitidis* HY0001					-	-	-	-	-	-	-	-	-	-	-

^a^ CSF, cerebrospinal fluid; N, nasopharyngeal swab; B, blood; RT, respiratory tract specimen; S, sputum; U, unknown.

^b^ +, amplification after a 60 min incubation; -, no amplification after a 60 min incubation.

Capsular type of 54 reference strains was identified by Quellung reaction using type-specific pneumococcal antisera (Statens Serum Institute, Copenhagen, Denmark). Since serotypes 11A and 11E could not be discriminated by Quellung reaction, strain SP2760 was indicated as serotype 11A/E. The P01-10 isolate was obtained from the Asian Bacterial Bank (ABB) of the Asia Pacific Foundation for Infectious Diseases (APFID) in Korea. The T9233/128/68, 559/66, 3405/39, Biehl, Hammer, and Eddy nr. 73 isolates were obtained from Aarhus University in Denmark.

### Preparation of chromosomal DNA

Chromosomal DNA from the 55 strains was prepared using the Wizard^®^ Genomic DNA Purification Kit (Promega, Fitchburg, WI, USA) per the manufacturer’s recommendations. The concentration of chromosomal DNA was measured on a NanoDrop 1000 (Thermo Fisher Scientific Inc., Waltham, MA, USA). The genome copy number was estimated based on the molecular size of *S*. *pneumoniae* strain R6 (2.0 Mbp; GenBank accession number, NC_003098). Each DNA sample was diluted to 10^5^ DNA copies/reaction and used to evaluate the specificity of the assays. For the detection limit study, serial 10-fold dilutions of genomic DNA from PPSV23 serotypes (capsular types 2 (D39), 8 (ATCC6308), 9N (SP2700), 10A (SP1933), 11A (SP2760), 12F (SP0113), 15B (SP3354), 17F (NCTC11904), 20 (SP2830), 22F (SP1854), and 33F (SP3201)) were amplified by LAMP, after which we compared the results with those of conventional PCR [27–30]. To determine the detection limit, duplicate LAMP testing of *S*. *pneumoniae* was carried out using serial 10-fold dilutions of chromosomal DNA over a 2-day period by two technicians. Ten-fold dilutions of each serotype of *S*. *pneumoniae* genomic DNA were amplified using the established LAMP and conventional PCR assays [27–30].

### LAMP primer design

Eleven LAMP primer sets for *S*. *pneumoniae* were designed based on the published sequences in GenBank and using LAMP primer design software (Table 2) [31]. A LAMP primer set consisted of 2 outer primers (F3 and B3), 1 forward inner primer (FIP), 1 backward inner primer (BIP), and loop primers (LF and/or LB).

**Table 2:** LAMP primer sequences in this study.

Primer name	LAMP primer sequence (sequence 5’-3’)	Length (base pairs)	Gene/ GenBank no. / target serotypes	Reaction temp.
2_F3	GGT TTT ACT GCT ATT TTT GG	20	*wzy*/ CR931633/ 2	63°C
2_B3	CTA AAT TTA ACA CTC GGT TCT	21		
2_FIP	CAT CGT TTG TAT CCA TTT AAC TGC ATG CAA CAT TTC AAT CTT ATG GT	47		
2_BIP	TCC CAG TTC AAT ATT TCT CCA CTA CAT GGG ATA ACA ATT ACC AA	44		
2_LB	GCA TAT GTT TTG TTA CTA GC	20		
8_F3	TTG GCA TTT CAA TTT TAT CCT TC	23	*wzy*/ CR931644/ 8	64°C
8_B3	AAT AAG ACT GAT AAG ATA AGT AGC C	25		
8_FIP	GAC CCC AGA ATG TAT AAC CAA AAA AGT TAT TGA TAA TAT CCT GAC TGG ACG	51		
8_BIP	GTT TGG GAT CCT ATT TGG GGA TTG CCA GCA TTA CTC ATC A	40		
8_LF	GTT CTT GCG TAT GCA GCT	18		
8_LB	ACT AGT TTC ACT TTT GAT TCG TT	23		
9N_F3	GAA TAT GGT ATC GTA GCA GTA G	22	*wzx*/ CR931647/ 9N, 9L	63°C
9N_B3	ACA CAA AGA CCT AAA AGC GG	20		
9N_FIP	TGC TGA TTA AAT CTG ACT TAT TCA GTT TCA AAT GTT AGC AGA TTC AGG	48		
9N_BIP	AGT GGA ATC GTA CTT TCT TGT ATT TAC TAA CAT AAA TAG TTT CGC CGT	48		
9N_LF	TTT GAA CAA TAG CAG GTC CT	20		
9N_LB	GGA TAT CCT ATG GGG GTG TT	20		
10A_F3	CTG TTG GAT TAT AAG GAA CAT GA	23	*wcrG*/ CR931649/ 10A	63°C
10A_B3	CAC CTG ATA ACA AAT GGA ACA T	22		
10A_FIP	ACT GGA CTT AAG CGA AGT AAT TGT TTT TAT TAT TGT AGG CAG CAA AG	47		
10A_BIP	TGT CAT TCT CGT AGA TAG AGT TCC TAT GCA TAA CGA AAT CCT AAC ATC	48		
10A_LF	AGA CTC CCC CAC ATT	15		
10A_LB	TGG GGA AAT TAT TCA CTA AG	20		
11A_F3	GAA GGA AGA TAT CTG TTG TAA CT	23	*wzy*/ CR931653/ 11A, 11D	63°C
11A_B3	CAA CTT CTC CCA ATT TCT GC	20		
11A_FIP	GGT ATC GAC CAT TAA AAG TAA AAT CTC TGT TGT GAT GTT AGC ATT AG	47		
11A_BIP	TGG CGC ATT GTG TAT GCT AAC GGA AGT TTA AAT TGA AAG CC	41		
11A_LF	CTA AGA TAT GGT AAA AAA TAT CCA	24		
11A_LB	CCA TTC TTC AAG TGA AAT GGT TTG	24		
12F_F3	ACA CCA TAT GAC TCC TTC T	19	*mnaB*/ CR931660/ 12F, 12A, 44, 46	65°C
12F_B3	CCC TGG AAT AAT ACG TTC TG	20		
12F_FIP	CGG TCG ATT CAA TAA TAA TAA CCG CGC TAT GTG ATT GAA GCT ACG	45		
12F_BIP	ATC ACC AGG AAC GGT TGA TAA ATG GAC AAG ATG AAT ATC ACT ACC	45		
12F_LF	GCA ATT ATC AAG TAC CGT T	19		
12F_LB	TTA TTC GAC CTG TTG TAG A	19		
15B_F3	CTA TGT TCA AAG AGG CGC	18	*wzy*/ CR931665/ 15B, 15C	63°C
15B_B3	AAG CAA GAA TAG TTA CTT GTG TA	23		
15B_FIP	CGT AAC TGG TAG CTG ATA CGA ATC AAT GTA GTA TTG TTT GGA AG	44		
15B_BIP	TGC AGG ATT TTT AGA ATA TTC AAC AAG TTT AAA TAA CCT AAT TAA CGG	48		
15B_LF	CGT CCC AAC CTA ATA AGC	18		
15B_LB	TAA ATG GGC AGT TGA TTC	18		
17F_F3	GAA TTA TTT GAA ATC TTA CCC CTC	24	*wciP*/ CR931670/ 17F	63°C
17F_B3	GCT AAT TCA TCA ATT TTC GAG AT	23		
17F_FIP	CTT TCA ATG CAG CAA TTT TTG TTG TCT TCC ATG TTA TGC TCC AGA	45		
17F_BIP	AGA CGA TAT GGG CAT AAT GTT ACA AGT TTT AAG ATT CGC TTC AAT GT	47		
17F_LF	CAT GTG ATA GGA AGG C	16		
20_F3	GAT AAG GTC TAC TTT GTG GGA	21	*wciL*/ CR931679/ 20	63°C
20_B3	CTT CAC TTT CCT TCA ATG GAT	21		
20_FIP	TCC TAA ACC TTC AAA ATT AGA CGG AGT TCA GTC GAA TAT ATC AGA ATG G	49		
20_BIP	CTG CAT TAG AGG CTC AAG TAA ATG GTG ATT TTT ACT TCC TTT GGT ACA	48		
20_LF	AGA ATA AGT CAA AAG TAC TC	20		
20_LB	CCG ACA CTA CTC TCA GA	17		
22F_F3	GGA TGA GGG TAA TAT TGT TGA	21	*wcwV*/ CR931682/ 22F, 22A	63°C
22F_B3	CTT GAA TCA GTG TCT CTC CT	20		
22F_FIP	TGG GTA AAT AAA TTG AAG CTG AGA GAC TAT TCA CGG TTT AAA GAT GA	47		
22F_BIP	TTG TGC TCC CGT CGA TTC AAA ACA GAC AAC TTA AAA TAC ATT GCC	45		
22F_LF	TCT TTA AAT AAG GTT GAT AA	20		
22F_LB	TTG GTA TGG TAT TAC TTG A	19		
33F_F3	CCC CAA CGG TTT ATG TGT T	19	*wzy*/ CR931702/ 33F, 33A, 37	63°C
33F_B3	TCC AAG TTG TGA AAT TCC ATT	21		
33F_FIP	CGC CCC CAA AAT CTC TAT TTT TTG TGT GGG GGA ATA GTA GAA GG	44		
33F_BIP	ACG GGG ATT TTT GCT GAA GCA GTT TGA AAT ATC AAT GCA AGG C	43		
33F_LF	GCT TCA AAA TGA AGA TTA TAG TAC	24		
33F_LB	CCT ATG TGG AGT TTG GTA TTG	21		

### LAMP reaction

The LAMP procedure has been described previously [25]. Briefly, we performed LAMP in a reaction mixture consisting of 1.6 μM each of FIP and BIP, 0.2 μM each of F3 and B3, 0.4 μM of LF/LB, 8 U of *Bst* DNA polymerase large fragment (New England Biolabs, Ipswich, MA, USA), 1.4 mM deoxynucleoside triphosphates, 0.8 M betaine (Sigma, St. Louis, MO, USA), 20 mM Tris-HCl (pH 8.8), 10 mM KCl, 10 mM (NH_4_)_2_SO_4_, 8 mM MgSO_4_, 0.1% Tween 20, and template DNA. The final volume was adjusted to 25 μL with distilled water. We incubated each reaction mixture at 63°C (serotypes 2, 9N, 10A, 11A, 15B, 17F, 20, 22F, and 33F), 64°C (serotype 8), or 65°C (serotype 12F) for 60 minutes and then heated it at 80°C for 2 minutes to terminate the reaction.

### Analysis of LAMP products

The turbidity of the reaction tube was determined in real time by optical density at 650 nm (OD_650_) in 6-s intervals using 2 Loopamp^®^ real-time turbidimeters (LA-500 and LA-200; Eiken Chemical Co., Tokyo, Japan). We calculated the amplification time required to exceed a turbidity of 0.1 (*Tt*) using turbidimeter software, as described [32].

Each amplified LAMP product was sequenced at the Akita Prefectural University Biotechnology Centre using the BigDye^®^ Terminator V3.1 cycle sequencing kit (Applied Biosystems, Foster City, CA, USA) on a 3130xL Genetic Analyser (Applied Biosystems), and their sequences were verified using the primers in S1 Fig.

We also tried to measure the detection using a colorimetric visual inspection dye (leucotriphenylmethane [33]; D-QUICK, Kaneka Co., Osaka, Japan) on an in-house DNA chip and mobile software. The color change on the DNA chip was examined using the mobile application.

### PCR assay

PCR was performed in 25-μL reaction mixtures, containing 1 U Ex Taq DNA polymerase (TaKaRa Bio, Tokyo, Japan), 0.2 mM of each deoxyribonucleoside triphosphate, 10 mM Tris-HCl buffer (pH 8.3), 50 mM KCl, 2 mM MgCl_2_, 0.5 μM of each primer, and 2 μL of template DNA, on a SimpliAmp^TM^ Thermal Cycler (Applied Biosystems, Foster City, CA, USA). The program comprised 35 cycles of denaturation at 94°C for 30 s, primer annealing at 54°C for 90 s, and extension at 72°C for 60 s, with a final incubation at 72°C for 10 minutes [27–29]. Previously reported PCR primers were used for detecting capsular types 2 and 8 [28], 9N [29], 10A, 11A, 12F, 15B, 17F, 20, 22F, and 33F [27] (S1 Table). The PCR products were electrophoresed on agarose gels and visualized with Midori Green Advance (NIPPON Genetics, Tokyo, Japan).

### DNA-spiked clinical blood specimens

Pneumococcal serotypes often are examined using blood samples, as they can be collected non-invasively, and a positive result indicates an IPD [34]. Blood was collected from 5 healthy volunteers and heparinized for storage. Using the Procedure for Ultra Rapid Extraction (PURE; Eiken Chemical, Tokyo), DNA from the blood samples was prepared and used for DNA-spiked blood experiments.

### Ethical declaration

We received written informed consent prior to collecting blood from 5 healthy volunteers. The procedures were approved by the IRB of Nihon University School of Medicine (IRB No. 28-9-5). All experiments were performed in accordance with the relevant guidelines and regulations.

## Results

### Analytical reactivity and specificity of LAMP-based pneumococcal serotyping

We established LAMP primer sets targeting the same genes as in successful conventional multiplex PCR-based assays [27–29]. The LAMP primer sets for capsular types 2, 8, 9N, 10A, 11A, 12F, 15B, 17F, 20, 22F, and 33F (Table 2) successfully amplified the target DNA sequence of each target locus (Table 1). We evaluated the analytical reactivity and specificity of LAMP-based pneumococcal serotyping using two non-pneumococcal species and 55 pneumococcal strains that belonged to 44 pneumococcal serotypes (Table 1). For each strain, a genomic DNA concentration of 10^5^ copies per reaction was used as a standard. No LAMP primer set amplified DNA of the two non-pneumococcal species. LAMP primer sets for capsular types 2, 8, 10A, 17F, and 20, which are single-serotype serogroups, did not generate any amplicon from the DNA of other target capsular types.

The LAMP primer set for capsular type 9N also amplified the DNA of capsular type 9L. DNA of other capsular types, including 9V, was not amplified. The pneumococcal capsular polysaccharide (CPS) genes of serogroup 9 could be grouped into 2 clusters based on sequence similarity; 9A and 9V had similar sequences [35], and 9N and 9L shared homologous sequences [36]. The flippase genes (*wzx*) of capsular types 9N and 9L, which were targeted for the LAMP reaction, had identical sequences and were detected by LAMP reaction. The DNA sequence of serotype 9V, which is included in PCV7, differed from serotypes 9L and 9N; thus, serotype 9V was not detected by the LAMP reaction for serotype 9N.

The LAMP primer set for capsular type 11A amplified DNA of capsular type 11D because these types have the same sequence for the oligosaccharide repeat unit polymerase (*wzy*) gene [36]. The DNA of other capsular types was not amplified.

Regarding the target gene of the conventional PCR primer set for capsular type 12F, Pai et al. [27] described *wzx* as the target gene. However, we confirmed they actually used the UDP-N-acetylmannosamine dehydrogenase MnaB (*mnaB*) gene as the target. Thus, in this study, we designed a LAMP primer set for 12F targeting of the *mnaB* gene. The LAMP primer set for capsular type 12F amplified DNA of capsular types 12A, 44, and 46 as they all have the same sequence for the *mnaB* gene. DNA of other capsular types was not amplified [36].

The LAMP primer set for capsular type 15B amplified DNA of capsular types 15B and 15C. DNA of other capsular types, including 15A, was not amplified. Capsular types 15B and 15C have the same sequence for the serotype 15B/15C-specific *wzy* gene, but the genes of 15A and 15F belong to a different *wzy* homolog group [36].

The LAMP primer set for capsular type 22F, targeting the *wcwV* gene of serotype 22F, detected capsular types 22A and 22F. DNA of other capsular types was not detected by this primer set. Capsular types 22A and 22F have the same sequence for the putative glycosyl transferase (*wcwV*) gene [36].

The LAMP primer set for capsular type 33F, targeting the *wzy* gene of serotype 33F, detected capsular types 33A, 33F, and 37. DNA of other capsular types was not detected by this primer set. Capsular types 33A, 33F, and 37 have the same sequence for the *wzy* gene.

Conventional multiplex PCR-based assays are not able to differentiate capsular types 9N, 11A, 12F, 22F, and 33F from their related pneumococcal serotypes because the same target genes are used by the LAMP and multiplex PCR assays. Here, the Quellung reaction can discriminate serotypes 9N, 11A, 12F, 22F, and 33F from their related pneumococcal serotypes. This is a limitation of the two molecular serotyping assays.

Amplified LAMP products were subjected to DNA sequencing to verify the specificity of the reaction (between F1 and B1; S1 Fig), and all LAMP assays produced serotype-specific products (S2 Fig).

### Detection limit of LAMP-based pneumococcal serotyping method

The detection limit of the LAMP assay was 100 genome copies per reaction. The detection limit of the PCR assay was 10^3^ genome copies per reaction for capsular types 8, 9N, 11A, 15B, and 33F; 10^4^ genome copies per reaction for capsular types 10A, 17F, and 20; 10^5^ genome copies per reaction for capsular type 12F; and 10^6^ genome copies per reaction for capsular type 22F (Table 3). The sensitivity of the LAMP assay was 10- to 10,000-fold greater than that of PCR-based pneumococcal serotyping. The products were inspected visually by monitoring the turbidity of the reaction reagent by real-time turbidimetry (S3 Fig). The detection limits of serotyping LAMP assays were identical for real-time measurement and direct visual inspection. No LAMP amplification was detected in control samples that lacked target DNA. The experiments were repeated in duplicate over 2 days, and identical results were obtained by 2 individuals.

**Table 3:** Detection limits of LAMP and PCR assays for DNAs of pneumococcal serotypes and using DNA-spiked blood specimens.

*S*. *pneumoniae*	Detection limit (Purified DNA)		Detection limit (DNA-spiked blood)	
serotypes	PCR^a^^,^^b^	LAMP^a^	PCR	LAMP
2	10^4^ copies^c^	10^2^ copies	10^4^ copies	10^2^ copies
8	10^3^	10^2^	10^3^	10^2^
9N	10^3^	10^2^	10^4^	10^2^
10A	10^4^	10^2^	10^6^	10^2^
11A	10^3^	10^2^	10^4^	10^2^
12F	10^5^	10^2^	10^6^	10^2^
15B	10^3^	10^2^	10^5^	10^2^
17F	10^4^	10^2^	10^6^	10^2^
20	10^4^	10^2^	10^4^	10^2^
22F	10^6^	10^2^	>10^6^	10^3^
33F	10^3^	10^2^	10^3^	10^2^

^a^ PCR results were obtained by electrophoretic analysis. LAMP results were determined visually.

^b^ Conventional PCR (serotypes 2 and 8 [28]; serotype 9N [29]; serotypes 10A, 11A, 12F, 15B, 17F, 20, 22F, and 33F [27]).

^c^ Number of genome copies per reaction.

### LAMP analysis of DNA-spiked specimens

Using DNA-spiked blood specimens, 2, 8, 9N, 10A, 11A, 12F, 15B, 17F, 20, and 33F were detected by LAMP assay with sensitivity as high as when using purified DNA as the template. The detection limit of the LAMP assay for serotype 22F decreased slightly from 100 copies to 10^3^ copies (Table 3).

The detection limits of the PCR-based pneumococcal serotyping assay for serotypes 2, 8, 20, and 33F were the same as when using purified DNA as the template. The detection limits for serotypes 9N and 11A decreased from 10^3^ to 10^4^ genome copies per reaction, and those for serotypes 10A and 17F decreased from 10^4^ to 10^6^ genome copies per reaction. The detection limit for serotype 12F declined from 10^5^ to 10^6^ genome copies per reaction, that for serotype 15B decreased from 10^3^ to 10^5^ genome copies per reaction, and that for serotype 22F decreased from 10^6^ to >10^6^ genome copies per reaction.

### LAMP assay using colorimetric dye and mobile software

**Fig 1:**
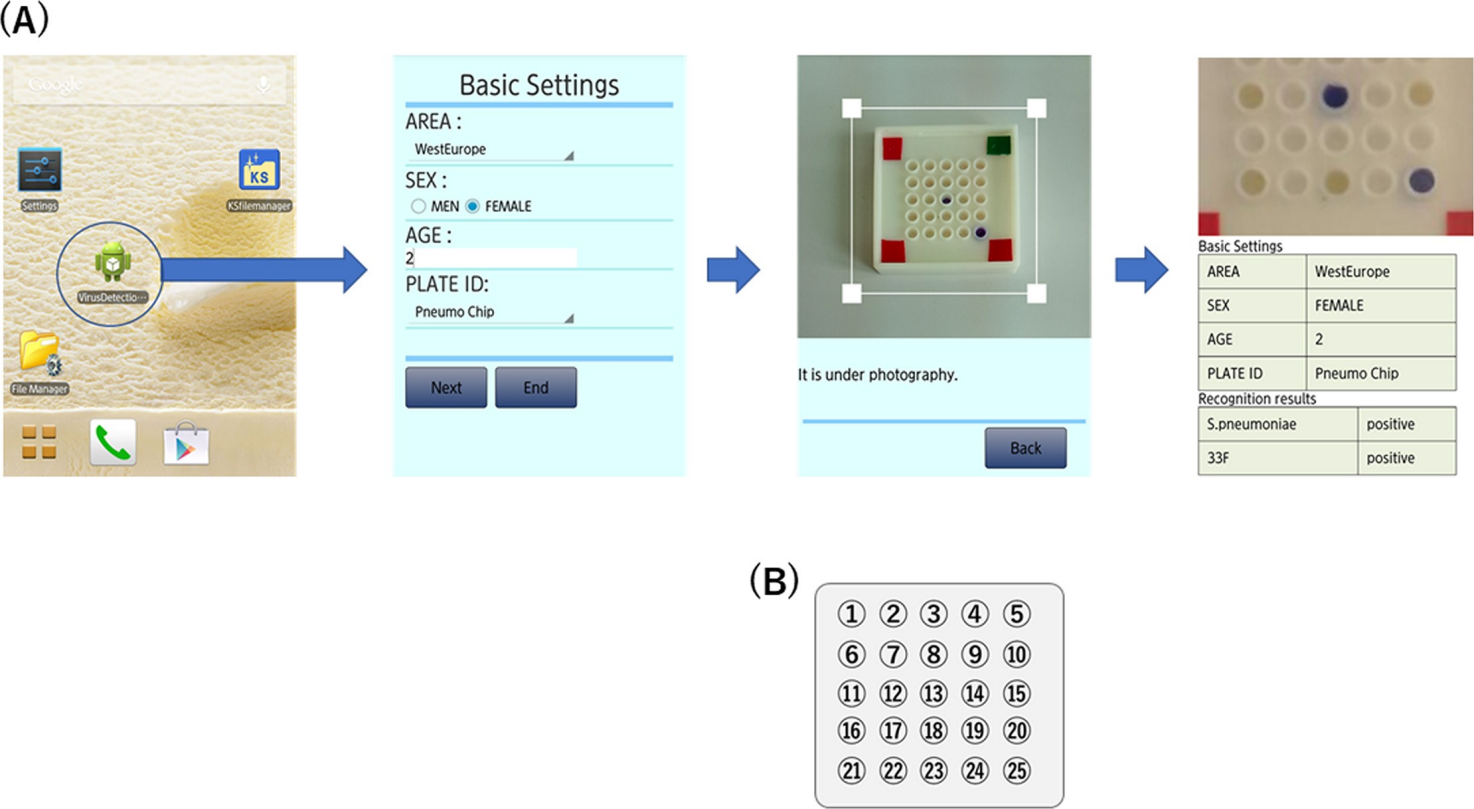
Flow chart of the LAMP assay using colorimetric visual inspection dye and mobile software. The color change of the LAMP reagents was observed and noted by the mobile software. (A) The color change of the LAMP reagents was observed and noted by the mobile software. (B) Target capsular types on the chip were as follows: 4, 6B, 9V, 14, 18C, 19F, 23F, 1, 3, 5, 7F, 19A, *S*. *pneumoniae lytA* gene as a positive control [21], 2, 6A (N/A)*, 8, 9, 10A, 11A, 12F, 15B, 17F, 20, 22F, and 33F in numerical order. *, the primer set for capsular type 6A is under development.

The results of the LAMP assay using a colorimetric dye and mobile software are shown in Fig 1. A color change in LAMP reagents was observed and successfully noted by the mobile software. The DNA chip and mobile software performed multiple and simultaneous LAMP detection of PPSV23, representing a novel advance for multiple and simultaneous LAMP detection.

## Discussion

PCVs have been introduced into the national immunization programs of most countries, and recent estimates suggest that approximately 100,000 deaths are prevented annually around the world [7]. The impact of PCVs on reducing antimicrobial-resistant pneumococcal strains has been reported [7, 13, 37]. Following PCV7 or 13 vaccination programs, diseases caused by *S*. *pneumoniae* serotypes that were not included in the vaccines (such as serotypes 8, 11A, 15A, 15B/C, 22F, 23A, 23B, and 24F) have been increasing in incidence [9, 38]. Japan experienced invasive pneumococcal diseases caused by serotypes 15A, 24F, and 35B, which are not targeted by the vaccine [39–41]. Approximately 80% to 90% of serotypes 35B and 15A are not susceptible to penicillin. The increased prevalence of non-vaccine serotypes that are highly non-susceptible to penicillin is a concern. The incidence of invasive pneumococcal serotype-specific disease should be monitored, and clinical and epidemiological characteristics must be understood.

Despite its importance, information on the burden of pneumococcal disease is limited, especially regarding the disease in older children and adults in low- and middle-income countries. Effective and easily accessible serotype identification tests are needed to ensure the impact of PCVs/PPSV23 and emergence of non-vaccine serotypes.

The Quellung reaction is the conventional serological typing method for pneumococcus, but its use is limited, especially in low- and middle-income countries [16]. Multiplex PCR-based assays for pneumococcal serotyping successfully identified pneumococcal serotyping but requires special equipment (thermocyclers/electrophoresis) and personnel to perform PCR assays. In addition, PCR results can be unclear.

The principle of LAMP is similar to that of PCR but uses a DNA polymerase with high strand displacement activity (*Bst* DNA polymerase). Thus, unlike in PCR, a DNA denaturation step is not needed in LAMP. LAMP amplification can occur at constant temperature (60–65°C); LAMP does not require a thermal cycler and the reaction can be verified by observation of the reaction mixture (no need for electrophoresis). While two primer regions are used by PCR, four types of LAMP primers covering six primer regions are used, two inner (FIP and BIP) and two outer primers (F3 and B3). Thus, the assay is extremely specific. Two loop primers can be added to accelerate the amplification reaction, generating 10^9^ times amplified products within 1 hour. The DNA sample preparation can be simplified because of the reaction robustness on LAMP. Due to high specificity, sensitivity, and robustness of the reaction, LAMP is more suitable for low-resource settings and field conditions than is PCR [42].

In this study, LAMP assays to discriminate 11 serotypes (2, 8, 9N, 10A, 11A, 12F, 15B, 17F, 20, 22F, and 33F) of *S*. *pneumoniae* in PPSV23 were established. The reaction specificity and sensitivity were validated under standard laboratory conditions, and the LAMP reactions were evaluated in clinical specimens to ensure the effectiveness of the assays in clinical application. The established LAMP assays performed better than PCR-based assays. The LAMP assays were 10–10^4^ times more sensitive than conventional PCR with purified DNA as a template, consistent with previous studies [20–22, 24].

To examine clinical application of the pneumococcal serotyping LAMP assay, we evaluated the sensitivity of the method using DNA-spiked blood specimens as a template. The pneumococcal serotype-specific LAMP reaction had nearly equal sensitivity with DNA-spiked blood samples when using purified DNA as a template. While PCR was inhibited by substances in the blood, such as heparin and other blood components, including heme, leukocyte DNA, and immunoglobulin G, LAMP reactions were not inhibited or were inhibited only slightly when using DNA-spiked blood [43]. The LAMP assay can be performed using simple DNA preparation methods and tolerates potentially disruptive biological elements (e.g., reaction inhibitors) better than PCR [44, 45].

LAMP reactions have been performed individually for each target, and the increase of turbidity of the reaction tube was used as the indication of positive results. However, the LAMP method is remarkably effective when combined with the DNA-chip technique, and the LAMP reaction chip allows multiplex and simultaneous detection [42, 46]. PCR requires thermocycling that induces processes such as evaporation and droplet collision and fusion on the chip. Thus, PCR, unlike LAMP, is not easy to apply in a DNA-chip and requires complex workflows and multiple separate devices and instruments [47]. Moreover, LAMP has potentially better specificity than PCR because of the use of multiple primer sets for the target sequences. False positives can be decreased compared to the PCR method.

In this study, LAMP products were observed by a color change, from colorless to violet, due to triphenylmethane dye, which binds double-stranded DNA (D-QUICK; Kaneka Co., Osaka, Japan), indicating a positive reaction [33]. The newly created mobile and original code technology recognizes the positive reaction on the chip. As shown in Fig 1, color changes on the DNA chip can be observed simultaneously, like the QR coding system of a mobile phone. These mobile phone technologies are a critical platform for improving access in low- and middle-income countries, and the coding system (e.g., bar-coding or QR coding) can run on existing phones globally. Such mobile phone software applications would enable rapid reporting of the test results and contribute to disease control measures.

Neither LAMP nor conventional PCR-based assays can differentiate capsular types 9N, 11A, 12F, 22F, and 33F from their related pneumococcal serotypes because the two assays use the same target genes. On the other hand, the Quellung reaction can discriminate all of these. This is the limitation of the two molecular serotyping assays.

This study is the first report of a LAMP-based PPSV23 serotype-specific identification assay. The rapid and simple PPSV23-targeted serotype detection assay can be used in many countries.　We are preparing further LAMP-based methods for non-vaccine-type targets. This study reports progress in development of LAMP-based methods that cover all pneumococcal serotypes.

## Supporting information

S1 Table PCR primers used in this study. (PDF)

S1 Fig Nucleotide sequences of the *S*. *pneumoniae* serotype-specific genes used to design the pneumococcal serotyping LAMP primers. The sequences used for LAMP primers are indicated by arrows. (a) to (k) in the figure are the sequences for *S*. *pneumoniae* serotype-specific genes for serotypes 2, 8, 9N, 10A, 11A, 12F, 15B, 17F, 20, 22F, and 33F, respectively.
(PDF)

S2 Fig Sequence data of amplified products of pneumococcal-serotype-specific LAMP. (a) to (k) in the figure are the sequences for the amplified products of *S*. *pneumoniae* serotype-specific genes for serotypes 2, 8, 9N, 10A, 11A, 12F, 15B, 17F, 20, 22F, and 33F, respectively.
(PDF)

S3 Fig Real-time monitoring of the LAMP-based serotype-specific method. (PDF)
