# Supplementary material for: 23-valent polysaccharide vaccine (PPSV23)-targeted serotype-specific identification of Streptococcus pneumoniae using the loop-mediated isothermal amplification (LAMP) method
Source: PLoS One. 2021 Feb 16;16(2):e0246699. doi: 10.1371/journal.pone.0246699 (PMC7886117; doi:10.1371/journal.pone.0246699)
Supplement: S1 Table — (PDF) [file pone.0246699.s001.pdf]

S1 Table. PCR primer sequences

primer name	PCR primer Sequence (sequence 5'-3')	Gene/ GenBank no.	Nucleotide position	Product size (bp)	Reference
2_F	TAT CCC AGT TCA ATA TTT CTC CAC TAC ACC	*wzy*/	10271	290	Carvalho et al. [28]
2_R	ACA CAA AAT ATA GGC AGA GAG AGA CTA CT	CR931633	10531		
8_F	GAA GAA ACG AAA CTG TCA GAG CAT TTA CAT	*wzy*/	11193	201	Carvalho et al. [28]
8_R	CTA TAG ATA CTA GTA GAG CTG TTC TAG TCT	CR931644	11364		
9N_F	GAA CTG AAT AAG TCA GAT TTA ATC AGC	*wzx*/	9966	516	Dias et al. [29]
9N_R	ACC AAG ATC TGA CGG GCT AAT CAA T	CR931647	10753		
10A_F	GGT GTA GAT TTA CCA TTA GTG TCG GCA GAC	*wcrG*/	12423	628	Pai et al. [27]
10A_R	GAA TTT CTT CTT TAA GAT TCG GAT ATT TCT C	CR931649	13020		
11A_F	GGA CAT GTT CAG GTG ATT TCC CAA TAT AGT G	*wzy*/	11640	463	Pai et al. [27]
11A_R	GAT TAT GAG TGT AAT TTA TTC CAA CTT CTC CC	CR931653	12071		
12F_F	GCA ACA AAC GGC GTG AAA GTA GTT G	*mnaB*/	14407	376	Pai et al. [27]
12F_R	CAA GAT GAA TAT CAC TAC CAA TAA CAA AAC	CR931660	14753		
15B_F	TTG GAA TTT TTT AAT TAG TGG CTT ACC TA	*wzy*/	7314	496	Pai et al. [27]
15B_R	CAT CCG CTT ATT AAT TGA AGT AAT CTG AAC C	CR931665	7779		
17F_F	TTC GTG ATG ATA ATT CCA ATG ATC AAA CAA GAG	*wciP*/	10484	693	Pai et al. [27]
17F_R	GAT GTA ACA AAT TTG TAG CGA CTA AGG TCT GC	CR931670	11145		
20_F	GAG CAA GAG TTT TTC ACC TGA CAG CGA GAA G	*wciL*/	9567	514	Pai et al. [27]
20_R	CTA AAT TCC TGT AAT TTA GCT AAA ACT CTT ATC	CR931679	10048		
22F_F	GAG TAT AGC CAG ATT ATG GCA GTT TTA TTG TC	*wcwV*/	11055	643	Pai et al. [27]
22F_R	CTC CAG CAC TTG CGC TGG AAA CAA CAG ACA AC	CR931682	11666		
33F_F	GAA GGC AAT CAA TGT GAT TGT GTC GCG	*wzy*/	11129	338	Pai et al. [27]
33F_R	CTT CAA AAT GAA GAT TAT AGT ACC CTT CTA C	CR931702	11436		
