# Supplementary material for: 23-valent polysaccharide vaccine (PPSV23)-targeted serotype-specific identification of Streptococcus pneumoniae using the loop-mediated isothermal amplification (LAMP) method
Source: PLoS One. 2021 Feb 16;16(2):e0246699. doi: 10.1371/journal.pone.0246699 (PMC7886117; doi:10.1371/journal.pone.0246699)
Supplement: S1 Fig — The sequences used for LAMP primers are indicated by arrows. (a) to (k) in the figure are the sequences for S. pneumoniae serotype-specific genes for serotypes 2, 8, 9N, 10A, 11A, 12F, 15B, 17F, 20, 22F, and 33F, respectively. (PDF) [file pone.0246699.s002.pdf]

S1 Fig. Nucleotide sequence of the *S. pneumoniae* serotype-specific genes used to design the pneumococcal serotyping LAMP primers. The sequences used for the LAMP primers are indicated by arrows. (a) to (k) in the figure are the sequences for *S. pneumoniae* serotype-specific genes for serotypes 2, 8, 9N, 10A, 11A, 12F, 15B, 17F, 20, 22F, and 33F, respectively.

Fig S1 (a). Target, serotype 2; GeneBank no. CR931633; Gene, *wzy*

```
No.        10021                                                       10080
sequence   AACAGTTATA TTCACACAAG ATTTATTGGT GATGACTTCT AAATTTATAT TATTTCCAAT

No.        10081                                                       10140
sequence   ATTTGTTTAT TATATAATAC CTAAAAATAT AAATGGCGTT ATAAGAATAC TATCTATTTT

No.        10141                                                       10200
sequence   TAAATCTTTC ATTGGTTTTA CTGCTATTTT TGGATTATAT GAATATATGC AACATTTCAA
primer                   |---------F3--------->               |---------F2--

No.        10201                                                       10260
sequence   TCTTATGGTG AATTTTGTAA AAATAGATGC AGTTAAATGG ATACAAACGA TGAATTTGAA
primer     -------->                    <-----------F1-------------|

No.        10261                                                       10320
sequence   TAGTGTGTAT TATCCCAGTT CAATATTTCT CCACTACACC TATTTTGCAT ATGTTTTGTT
primer                  |-----------B1------------>          |---------LB---

No.        10321                                                       10380
sequence   ACTAGCTTTT ATTTTGGTAA TTGTTATCCC ATATAAGAAC CGAGTGTTAA ATTTAGTTTA
primer     ----->        <---------B2--------|   <---------B3----------|

No.        10381                                                       10440
sequence   TAAAACACTT ATTGCAATTT CAATTTTTTT GCCCCAATCT CGTATAGTTT GGATAGCTTT

No.        10441                                                       10500
sequence   TGGGGTAATT TTAATTCTTT CGTTTATACT AAATAGACAG GGGATATTAA CCTATAGAAA

No.        10501                                                       10560
sequence   GTTGAGTGTT ATAGTACTTA TTTTAATTAT AGTAGTCTCT CTCTGCCTAT ATTTTGATGT
```

Fig S1 (b). Target, serotype 8; GeneBank no. CR931644; Gene, *wzy*

```
No.        11341                                                        11400
sequence   GTTATTCTTT TATTTTTTCA CGCAGACTAG AACAGCTCTA CTAGTATCTA TAGTAATTTT


No.        11401                                                        11460
sequence   TGCTCTTTTA TATATTTATA TGTTTGTTGA AAATCTTGAA CTTAGATGGA TAGGATACTC


No.        11461                                                        11520
sequence   ATTCTTTTGT ATTTCTACTT TTTTAGGAGT TTTGGCATTT CAATTTTATC CTTCTAATAA
primer                                       |-----------F3---------->

No.        11521                                                        11580
sequence   TAAGTTTTCA ATTTTTATTG ATAATATCCT GACTGGACGT ATTAAATTAG CTGCATACGC
primer                    |------------F2----------->          <--------LF--

No.        11581                                                        11640
sequence   AAGAACCTTT TTTGGTTATA CATTCTGGGG TCAATATGTT GATAAAGAAA TTGTTTGGGA
primer     -----|<------------F1-------------|                      |-------

No.        11641                                                        11700
sequence   TCCTATTTGG GGATTAACTA GTTTCACTTT TGATTCGTTT TATTCCTTCT TGATGAGTAA
primer     ---B1--------->  |----------LB----------->             <-------B2

No.        11701                                                        11760
sequence   TGCTGGCATC ATATGGCTAC TTATCTTATC AGTCTTATTT GTAAAACTAC AAAAATATTT
primer     -------|       <-----------B3------------|

No.        11761                                                        11820
sequence   AGACAATAAA AGTTTGATTT TATTATTAGC TTGGTCTATG TATGCGGTAA CAGAAACTGA


No.        11821                                                        11880
sequence   TTTAATATTT CCAAGTTATG GATTCCAGTT CTTATTTTTA AGTATACTTT TTACTAATAC
```

Fig S1 (c). Target, serotype 9N, 9L; GeneBank no. CR931647; Gene, *wzx*

```
No.        11701                                                       11760
sequence   TTGAATATTA TCAAATTAAT TTTACAGAGG ATAAATAATG AATGTTGAAA AAAATTTGAA


No.        11761                                                       11820
sequence   ACGTGGAATT TTCTATACTG CAATAGGGAA GTATTCGAAT GTAGTTATTC AATTACTTGT


No.        11821                                                       11880
sequence   AACAGCAATT CTTAGCCGGA TTCTCTCACC TGTAGAATAT GGTATCGTAG CAGTAGTAAA
primer                                          |----------F3----------->

No.        11881                                                       11940
sequence   TGTATTTCTT TTATTTTTTC AAATGTTAGC AGATTCAGGA ATAGGACCTG CTATTGTTCA
primer                      |-----------F2----------->   <---------LF-------

No.        11941                                                       12000
sequence   AAATAAAGAA CTGAATAAGT CAGATTTAAT CAGCATTTTC TCATTAACGA TTTATAGTGG
primer     -|         <-----------F1------------|                      |----

No.        12001                                                       12060
sequence   AATCGTACTT TCTTGTATTT TTGCTTTACT AGGATATCCT ATGGGGGTGT TATACGGCGA
primer     -------B1----------->              |---------LB---------> <------

No.        12061                                                       12120
sequence   AACTATTTAT GTTAGTTTAT TTCCGCTTTT AGGTCTTTGT GTTTTATTTT ATACCATTAC
primer     -----B2---------|       <---------B3---------|

No.        12121                                                       12180
sequence   AATTGTACCG CAAGCTATTC TAATGAAAAC GATGAACTTT AAAATAGTTA ATTTTCTAAC


No.        12181                                                       12240
sequence   TATTTTTTCT AACATTGCTA GTGGCTTGGT GGGCGTTATT CTAGCGGTAT CACATTTTGG
```

Fig S1 (d). Target, serotype 10A; GenBank no. CR931649; Gene, *wcrG*

```
No.        12061                                                       12120
sequence   TAGTATAGTA GATTAATGTA TTTGATAAGA ATATCGAGTA TTTTAAGAAA CGAGGAAATA


No.        12121                                                       12180
sequence   TGAAACGAGG AAATATGAAA CAGGCTTATA TAATAATTGC ACATAATAAG TTTGAACAGT


No.        12181                                                       12240
sequence   TGAAATTTTT AATTTCTCTG TTGGATTATA AGGAACATGA TATTTTTATT ATTGTAGGCA
primer                       |-----------F3---------->   |----------F2------

No.        12241                                                       12300
sequence   GCAAAGTTAA TGTGGGGGAG TCTACAATTA CTTCGCTTAA GTCCAGTGCA ATACATTCCA
primer     ----->  <------LF-------|<-----------F1-----------|

No.        12301                                                       12360
sequence   ATGTCATTCT CGTAGATAGA GTTCCTATTT ATTGGGGAAA TTATTCACTA AGTTCTGCAG
primer      |------------B1----------->       |---------LB--------->

No.        12361                                                       12420
sequence   AGATGTTAGG ATTTCGTTAT GCATATAATT ACGATAACTA TAGTATGTTC CATTTGTTAT
primer      <----------B2-----------|                      <----------B3----

No.        12421                                                       12480
sequence   CAGGTGTAGA TTTACCATTA GTGTCGGCAG ACAAATTATT TAAATTCTTT GATCAGAACA
primer     -----|

No.        12481                                                       12540
sequence   AATCAAATAA TTTTTTGAGC ATGGTCTCTG ATGAGATTTT GAAATCAAAT AAAGTGTATG


No.        12541                                                       12600
sequence   AGCGTGTAAA ATTTAGATAC TTATTTCCTA GATTCTTAGC TAGAAATATT CAGAACAAAT
```

Fig S1 (e). Target, serotype 11A, 11D; GenBank no. CR931653; Gene, *wzy*

```
No.        11701                                                       11760
sequence   TATTAGAAGA AAAGAGAAAT ATATTAGTGG CACAGTTGCT GTTGATTTTA ACGATTATCA


No.        11761                                                       11820
sequence   ATTGTTTTTT TTCAGATGTC TCTCTCTCTA AAGTTATTGC TTTGTTTATG ATTGTTTATA


No.        11821                                                       11880
sequence   TTATTTCATA CAAGTTAAGA AAACTATTCT GGAAAAGAGG AAGGAAGATA TCTGTTGTAA
primer                                               |-----------F3---------

No.        11881                                                       11940
sequence   CTTTTATTGC ATCTGTTGTG ATGTTAGCAT TAGTAATTTT TGGATATTTT TTACCATATC
primer     ->          |----------F2---------->        <-----------LF-------

No.        11941                                                       12000
sequence   TTAGATATTT TGATTTTACT TTTAATGGTC GATACCAAAT TTGGCGCATT GTGTATGCTA
primer     ---|        <------------F1-----------|      |---------B1------->

No.        12001                                                       12060
sequence   CCATTCTTCA AGTGAAATGG TTTGGATATG GTTTGTTTGG CTTTCAATTT AAACTTCCGT
primer     |-----------LB----------->               <----------B2----------|

No.        12061                                                       12120
sequence   GGCAGAAATT GGGAGAAGTT GGAATAAATT ACACTCATAA TCAAGTTTTA CAGTTAGCAC
primer      <---------B3---------|

No.        12121                                                       12180
sequence   TTGATAGTGG TATTGTTGGG ATTATCTCAT TCTTTACTAT GATTTTTTAT ATGATCTTTT


No.        12181                                                       12240
sequence   CAACTAAAAA TATTCAAAAT TCCACAATAA GCTCTCTATT TATTTTTGCC TATTTTTGTC
```

Fig S1 (f). Target, serotype 12F, 12A, 44, 46; GenBank no. CR931660; Gene, *mnaB*

```
No.        14461                                                       14520
sequence   ACTTTAAATG AAGGTCAAAC AACCTTTAAA GAAGATGGAT TGGATGAACT ATTCCATAAA


No.        14521                                                       14580
sequence   GCAGTGGAGT CGGGTGTGGA CTTTACAACC GAATATCAAC AAACGGATAC CTATATTATT


No.        14581                                                       14640
sequence   TCCGTTCCAA CACCATATGA CTCCTTCTCT AAAAAAATTG ATCCAAGCTA TGTGATTGAA
primer              |--------F3--------->                    |-------F2-----

No.        14641                                                       14700
sequence   GCTACGAAAA CGGTACTTGA TAATTGCAAT AAAGGAGCGG TTATTATTAT TGAATCGACC
primer     ----->  <--------LF---------|          <------------F1-----------

No.        14701                                                       14760
sequence   GTATCACCAG GAACGGTTGA TAAATTTATT CGACCTGTTG TAGAAGAAAA AGGTTTTGTT
primer     | |-----------B1---------->|---------LB-------->

No.        14761                                                       14820
sequence   ATTGGTAGTG ATATTCATCT TGTCCATGCT CCAGAACGTA TTATTCCAGG GAATATGGTC
primer        <-----------B2---------|       <---------B3---------|

No.        14821                                                       14880
sequence   TATGAATTGG TGAATAATAA CCGTACAATT GGAGCTGATG ACCTAGAAAT CGGCTATAAA


No.        14881                                                       14940
sequence   GTGAAAGAGC TTTATGCTTC ATTTTGTAAG GGCGATATTG TTGTTACAGA TATTAGAACT


No.        14941                                                       15000
sequence   GCGGAGATGA CCAAGGTAGT AGAAAATACA TTTAGAGCTG TAAATATTGC TTTCGCTAAC
```

Fig S1 (g). Target, serotype 15B, 15C; GenBank no. CR931665; Gene, *wzy*

```
No.        7321                                                           7380
sequence   TTTTTAATTA GTGGCTTACC TATTCAAGTG TTGTTTTCAG ATTTGAGTAA GGCATTCAAT


No.        7381                                                           7440
sequence   TGGATATTAG CAGTATTTTT TTATAATTAT TATTTGAAAA ATCCCATTAA CGTTGACAAG


No.        7441                                                           7500
sequence   ATAAAGAAAT ATATGTTTTA TAATTTCGCT ATATTAGTTA TTATTGTTGC TTTATTCTAT
primer                                                                    |---

No.        7501                                                           7560
sequence   GTTCAAAGAG GCGCTAATGT AGTATTGTTT GGAAGAAGCT TATTAGGTTG GGACGGATTC
primer     ----F3--------> |---------F2---------> <------LF----------|<----

No.        7561                                                           7620
sequence   GTATCAGCTA CCAGTTACGG AGTAAGATAT GCAGGATTTT TAGAATATTC AACATTAAAT
primer     ------F1-----------|           |------------B1------------> |----

No.        7621                                                           7680
sequence   GGGCAGTTGA TTCTTTTTTT GTTACCGTTA ATTAGGTTAT TTAAACTTAG TTTTTTTACA
primer     -----LB------>            <----------B2-----------|          <---

No.        7681                                                           7740
sequence   CAAGTAACTA TTCTTGCTTT TTTGCTACAG GTTTTAGTAT TGAGTAAATC TAGAATAGCT
primer     --------B3---------|

No.        7741                                                           7800
sequence   ATTATTGCTC TGATTATATA CATAGTATTT GTAGTAATGG TTCAGATTAC TTCAATTAAT


No.        7801                                                           7860
sequence   AAGCGGATGA TTGTAGCGTT TTATCCAACT ATACCTCTTA TATTGCTTTA TAATTGGGAA
```

Fig S1 (h). Target, serotype 17F; GenBank no. CR931670; Gene, *wciP*

```
No.        10741                                                    10800
sequence   CCGGTATTGA TTTACGCTGA TATGCGGATT ATAGATGCTA ACGGTAAAGT GATAGCTAAT


No.        10801                                                    10860
sequence   AGTATGGATC AATTGATGGG AATTCGGTAT ACCAATCCTA TCTCAACTTT TATGGCTCAT


No.        10861                                                    10920
sequence   AAGGTTTATG GATGTAATAC GTTATTTAAT CATGAATTAT TTGAAATCTT ACCCCTCCTT
primer                                        |-----------F3----------->|--

No.        10921                                                    10980
sequence   CCATGTTATG CTCCAGAATT AGCCTTCCTA TCACATGATA ACTTTACAAC AAAAATTGCT
primer     -------F2-------->     <------LF-------|         <-------F1------

No.        10981                                                    11040
sequence   GCATTGAAAG GGCATGTGTA TTTTTATGAT GAGCCTACTA TGAGTTATAG ACGATATGGG
primer     ---------|                                          |------B1----

No.        11041                                                    11100
sequence   CATAATGTTA CAAGTAAACA TGAGTATAAC TTTACATTGA AGCGAATCTT AAAACGTATC
primer     ------------->                      <----------B2----------|  <--

No.        11101                                                    11160
sequence   TCGAAAATTG ATGAATTAGC TAAAGATCAT GCCTTGACTT ACAAGCAGAC CTTAGTCGCT
primer     -------B3-----------|

No.        11161                                                    11220
sequence   ACAAATTTGT TACATCAACA AACAAGTATT GACACTGAAC TCCTATTTTT AGATAAGGTA


No.        11221                                                    11280
sequence   GAAAAAATAA TCAAAAAAGG TGGATTAAAT GCTGTAAAAA TGGTTTGGAA AGAAAAAATT
```

Fig S1 (i). Target, serotype 20; GenBank no. CR931679; Gene, *wciL*

```
No.        9961                                                         10020
sequence   TAAAATTCGA ATTGATTATA CATTAGACAA TAAATTTGTG CTAGGTCATG TAGGACGTTT

No.        10021                                                        10080
sequence   GCATTTTCAG AAGAATCAAG AATTTATGAT AAGAGTTTTA GCTAAATTAC AGGAATTTAG

No.        10081                                                        10140
sequence   AGACGATGTC TGCTTAGTTT TAGTTGGTCA AGGTGAAGAT CTTAACAAAT TAAAGATACT

No.        10141                                                        10200
sequence   GGCTGAGGAG CTATCTATTC AGGATAAGGT CTACTTTGTG GGAGTTCAGT CGAATATATC
primer                             |----------F3--------->|-----------F2----

No.        10201                                                        10260
sequence   AGAATGGTTG AGTACTTTTG ACTTATTCTT TTTTCCGTCT AATTTTGAAG GTTTAGGAAT
primer     ------>  <---------LF---------|     <-----------F1------------|

No.        10261                                                        10320
sequence   AGCTGCATTA GAGGCTCAAG TAAATGGTCT ACCGACACTA CTCTCAGAAG AAGGTGTACC
primer       |------------B1----------->     |--------LB------>       <-----

No.        10321                                                        10380
sequence   AAAGGAAGTA AAAATCAATG ATAATACTTT TTTTTATCCA TTGAAGGAAA GTGAAGAAAG
primer     ------B2---------|                    <---------B3----------|

No.        10381                                                        10440
sequence   CTGGGCAAAC TTCCTTGATG AAATGATTCA TACAACAACT CGTTTAAATT ATGAATATAT

No.        10441                                                        10500
sequence   ACAAGAAAAT TTTGAGAAAT CTGGCTACGA TATTAAGATA GCTGCTCAGA ATCTTGAAAA
```

Fig S1 (j). Target, serotype 22F, 22A; GenBank no. CR931682; Gene, *wcwV*

```
No.        11361                                                      11420
sequence   AAGCAAAATA TTCTTTATGT AGGCTCACTA TCAAAAAGAA AAAACACAGC TCATTTAATT

No.        11421                                                      11480
sequence   AGAATTTTTA ATATTTTAAA ATCTAAAAGT GGTAAGAAAA ACGAACTTCA ATTGGTATTG

No.        11481                                                      11540
sequence   ATTGGTAAGG ATGAGGGTAA TATTGTTGAA AAAATTAACT ATTCACGGTT TAAAGATGAT
primer             |----------F3--------->         |----------F2---------->

No.        11541                                                      11600
sequence   ATTATTTATC AACCTTATTT AAAGAACTCT CAGCTTCAAT TTATTTACCC ATCATCACAA
primer          <----------LF--------| <------------F1------------|

No.        11601                                                      11660
sequence   CTATTTGTGC TCCCGTCGAT TCAAGAGATT TTTGGTATGG TATTACTTGA GGCAATGTAT
primer         |----------B1-------->        |--------LB--------> <---------

No.        11661                                                      11720
sequence   TTTAAGTTGT CTGTTGTTTC CAGCGCAAGT GCTGGAGGAG AGACACTGAT TCAAGATGGT
primer     --B2-----------|                      <---------B3---------|

No.        11721                                                      11780
sequence   ATAAACGGTA AAATTATGAA TGATTTTAAT GATGAACACT GGGTGGATTG TATAGAAAAT

No.        11781                                                      11840
sequence   CTATTAAATA ACCCATTGGA ATTGAAACGG CTTGGAGAAT GTGCCCATAA GCGAATTACG

No.        11841                                                      11900
sequence   GAACAGTTTA TGTGGTCTTC AATTGCGAGA AAAATAATTG AGACATTTGA TGAAAGGTAA
```

Fig S1 (k). Target, serotype 33F, 33A, 37; GenBank no. CR931702; Gene, *wzy*

```
No.        11221                                                  11280
sequence   CAAGTTTCGT CTATTATTTT GTATTATTTC CGATTTTTAT GATGATTTTG CAGATGTACT


No.        11281                                                  11340
sequence   ATGATGTTAA TGAAATCGCA AATCTGATAC GGAAATTTGT TCGTATAATA TTTCTTTTAG


No.        11341                                                  11400
sequence   CAATTGGCTC TCTCCTATTT TGGCTTATTG GTAGTGTATT TCATATTATA TCCCCAACGG
primer                                                      |--------

No.        11401                                                  11460
sequence   TTTATGTGTT GAATTATTGG AATGGTGGGG GAATAGTAGA AGGGTACTAT AATCTTCATT
primer     F3------->                |---------F2--------><-----------LF----

No.        11461                                                  11520
sequence   TTGAAGCACA AAAAATAGAG ATTTTGGGGG CGATACTGAT AAGAAATACG GGGATTTTTG
primer     ------|<------------F1------------|                |---------B1--

No.        11521                                                  11580
sequence   CTGAAGCACC TATGTGGAGT TTGGTATTGA GCCTTGCATT GATATTTCAA ACGCTTCATA
primer     ------->|---------LB---------->  <----------B2----------|

No.        11581                                                  11640
sequence   TAAAAAAATG GAATTTCACA ACTTGGACTC TTATTATTAC AATTATGACA ACTACGTCAA
primer           <---------B3----------|

No.        11641                                                  11700
sequence   CAACAGGGGT TTATATAATA GGTTTGATTT TCCTATATGT TTTATTTTCA AAAACAAGTG


No.        11701                                                  11760
sequence   GTGTGAAGAG ATATGTTTCT AGTTTATTTA TTTTAGCGAT TATATGTTGT TTTTCAATAT
```
