# Supplementary material for: 23-valent polysaccharide vaccine (PPSV23)-targeted serotype-specific identification of Streptococcus pneumoniae using the loop-mediated isothermal amplification (LAMP) method
Source: PLoS One. 2021 Feb 16;16(2):e0246699. doi: 10.1371/journal.pone.0246699 (PMC7886117; doi:10.1371/journal.pone.0246699)
Supplement: S2 Fig — (a) to (k) in the figure are the sequences for the amplified products of S. pneumoniae serotype-specific genes for serotypes 2, 8, 9N, 10A, 11A, 12F, 15B, 17F, 20, 22F, and 33F, respectively. (PDF) [file pone.0246699.s003.pdf]

S2 Fig. Sequence data of amplified products of the pneumococcal-serotype-specific LAMP. (a) to (k) in the figure are the sequences for the amplified products of *S. pneumoniae* serotype-specific genes for serotypes 2, 8, 9N, 10A, 11A, 12F, 15B, 17F, 20, 22F, and 33F, respectively.

(a)

TGCAGTTAAA TGGATACAAA CGATGAATTT GAATAGTGTG
TATTATCCCA GTTCAATATT TCTCCACTAC

(b)

CTTTTTTGGT TATACATTCT GGGGTCAATA TGTTGATAAA
GAAATTGTTT GGGATCCTAT TTGGGGAT

(c)

CTGAATAAGT CAGATTTAAT CAGCATTTTC TCATTAACGA
TTTATAGTGG AATCGTACTT TCTTGTATTT

(d)

ACAATTACTT CGCTTAAGTC CAGTGCAATA CATTCCAATG
TCATTCTCGT AGATAGAGTT CCT

(e)

GATTTTACTT TTAATGGTCG ATACCAAATT TGGCGCATTG
TGTATGCTA

(f)

GCGGTTATTA TTATTGAATC GACCGTATCA CCAGGAACGG
TTGATAAAT

(g)

GATTCGTATC AGCTACCAGT TACGGAGTAA GATATGCAGG ATTTTTAGAA TATTCAACA

(h)

ACAACAAAAA TTGCTGCATT GAAAGGGCAT GTGTATTTTT ATGATGAGCC TACTATGAGT TATAGACGAT ATGGGCATAA TGTTACAA

(i)

TCCGTCTAAT TTTGAAGGTT TAGGAATAGC TGCATTAGAG GCTCAAGTAA ATGG

(j)

CTCTCAGCTT CAATTTATTT ACCCATCATC ACAACTATTT GTGCTCCCGT CGATTCAA

(k)

ACAAAAAATA GAGATTTTGG GGGCGATACT GATAAGAAAT ACGGGGATTT TTGCTGAAGC A
