# Supplementary material for: 23-valent polysaccharide vaccine (PPSV23)-targeted serotype-specific identification of Streptococcus pneumoniae using the loop-mediated isothermal amplification (LAMP) method
Source: PLoS One. 2021 Feb 16;16(2):e0246699. doi: 10.1371/journal.pone.0246699 (PMC7886117; doi:10.1371/journal.pone.0246699)
Supplement: S3 Fig — (PDF) [file pone.0246699.s004.pdf]

S3 Fig. Realtime monitoring of the LAMP based serotype specific method.

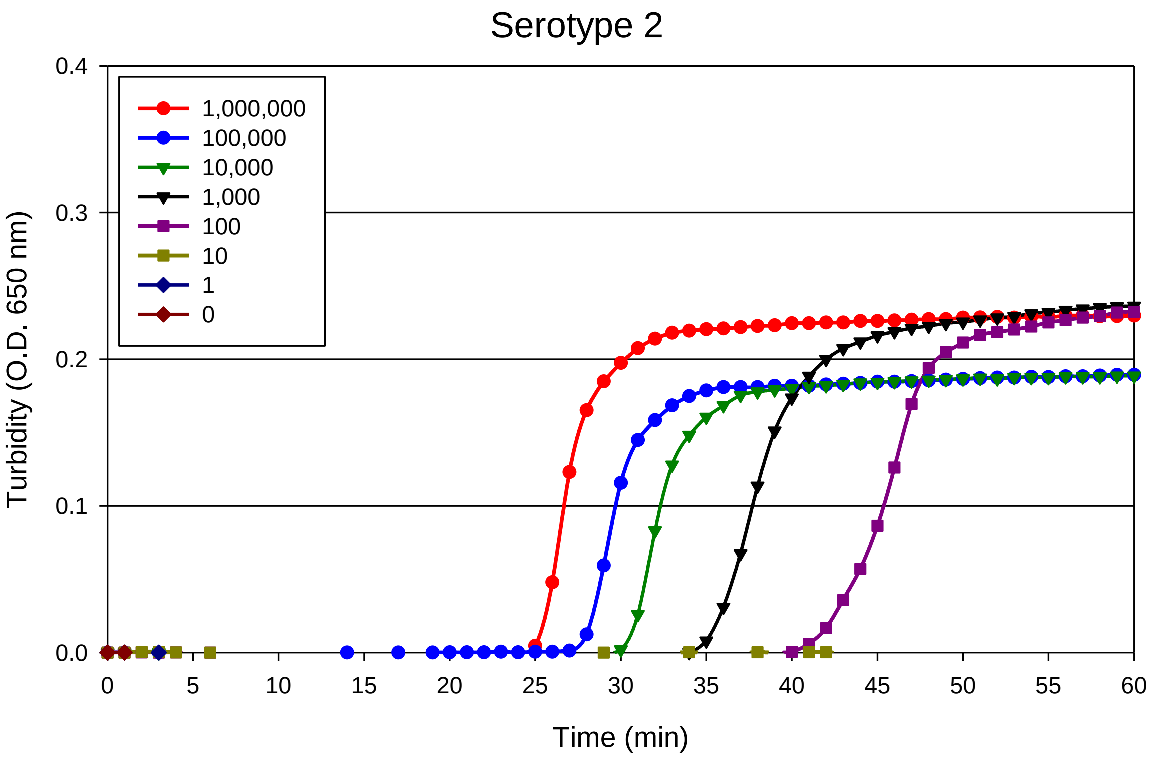

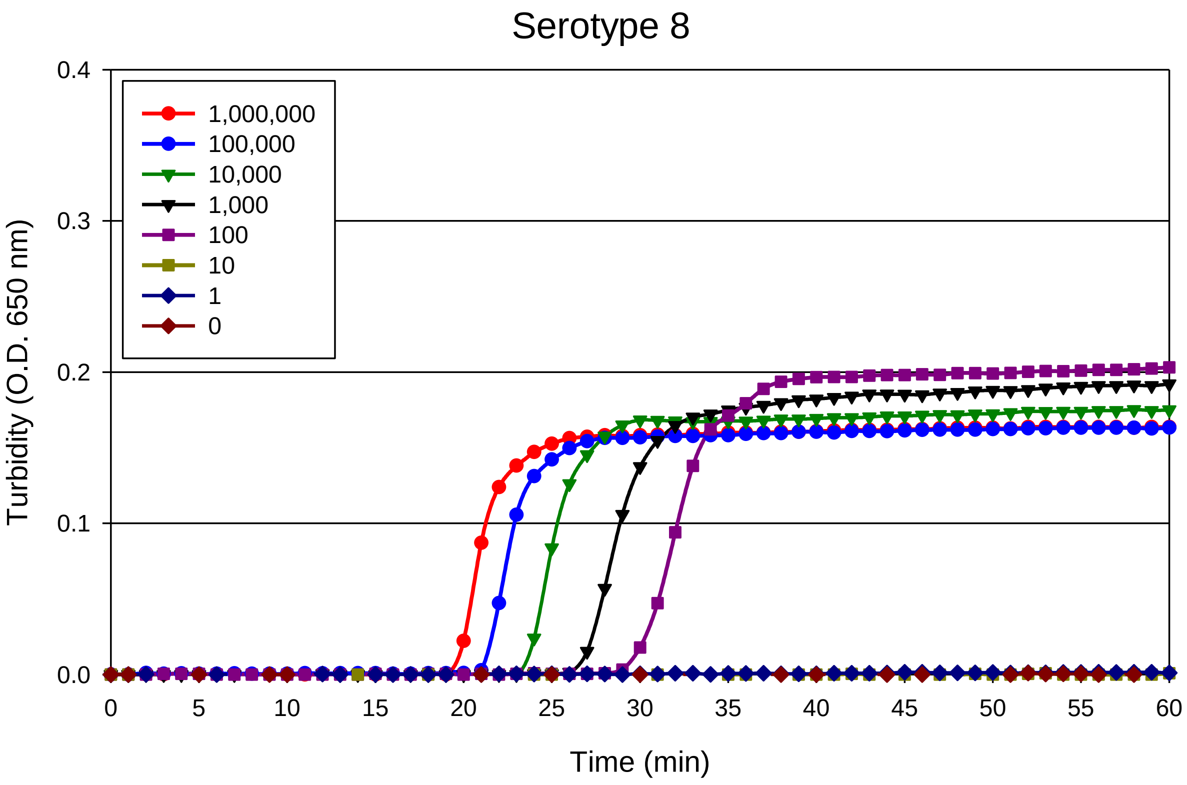

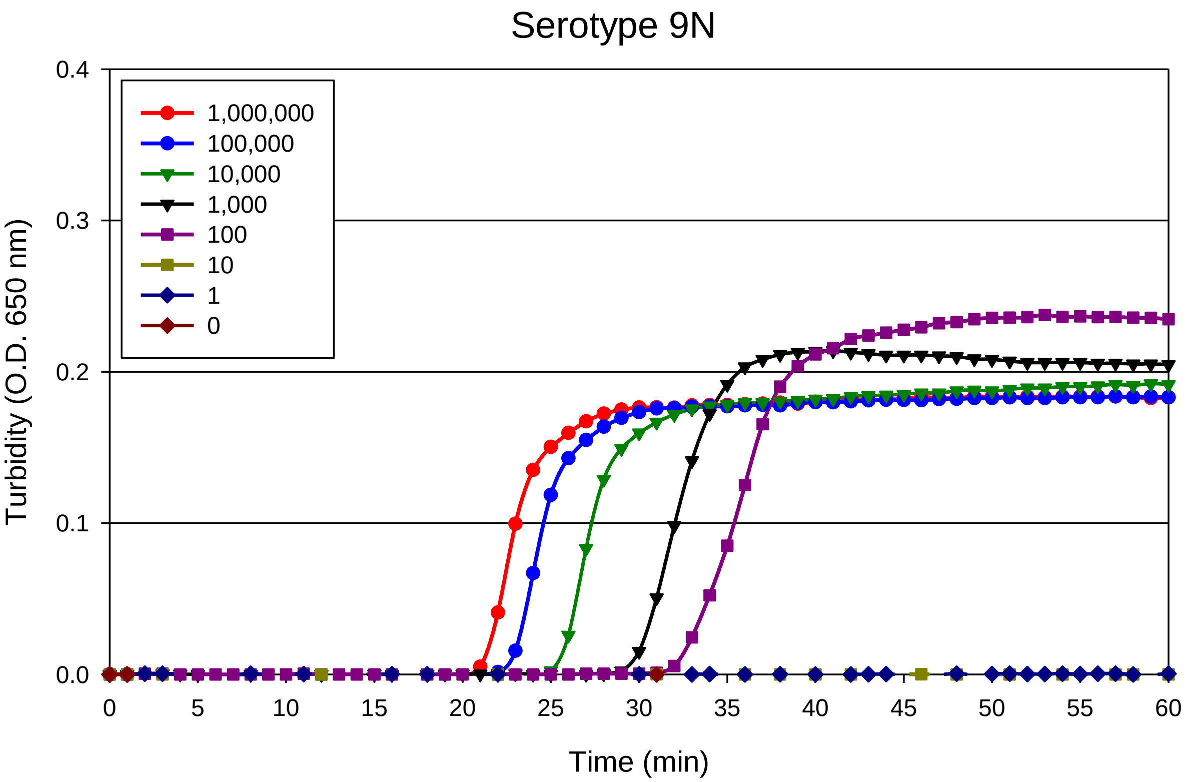
Serotype 9N
Turbidity (O.D. 650 nm)
0.4
0.3
0.2
0.1
0.0
0
5
10
15
20
25
30
35
40
45
50
55
60
Time (min)
1,000,000
100,000
10,000
1,000
100
10
1
0

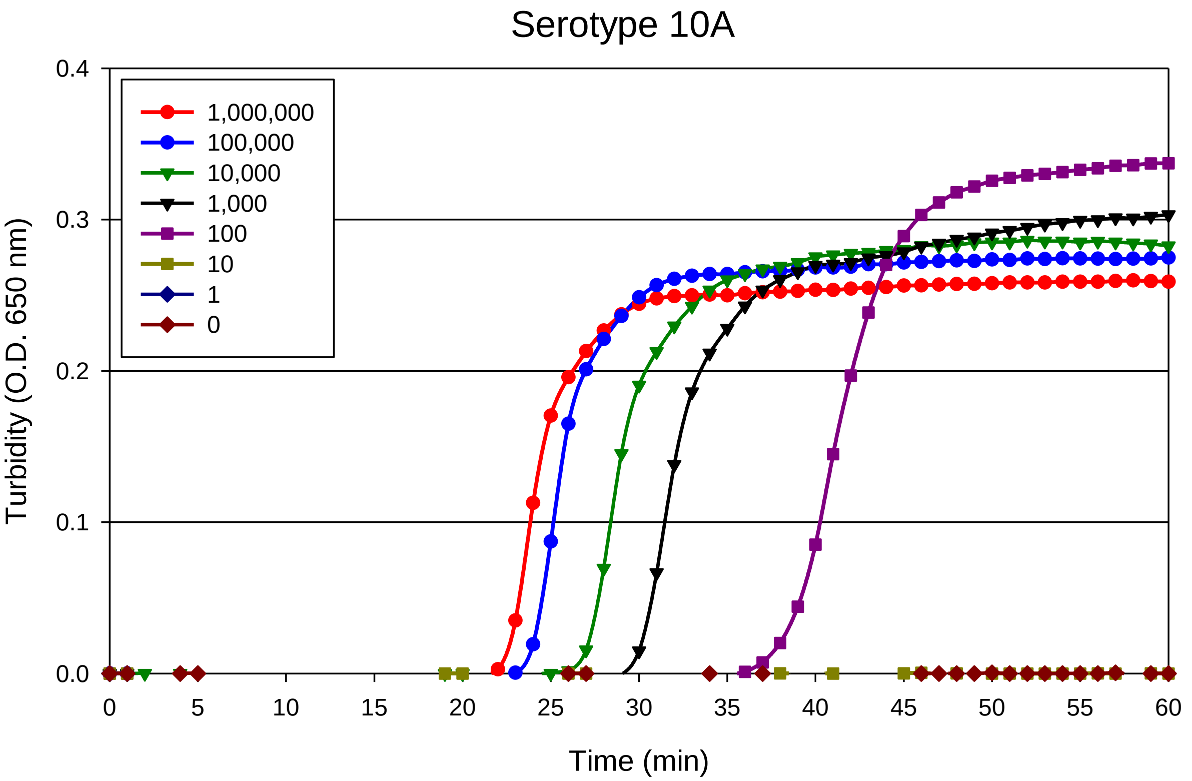
Serotype 10A
Turbidity (O.D. 650 nm)
0.4
0.3
0.2
0.1
0.0
0
5
10
15
20
25
30
35
40
45
50
55
60
Time (min)
1,000,000
100,000
10,000
1,000
100
10
1
0

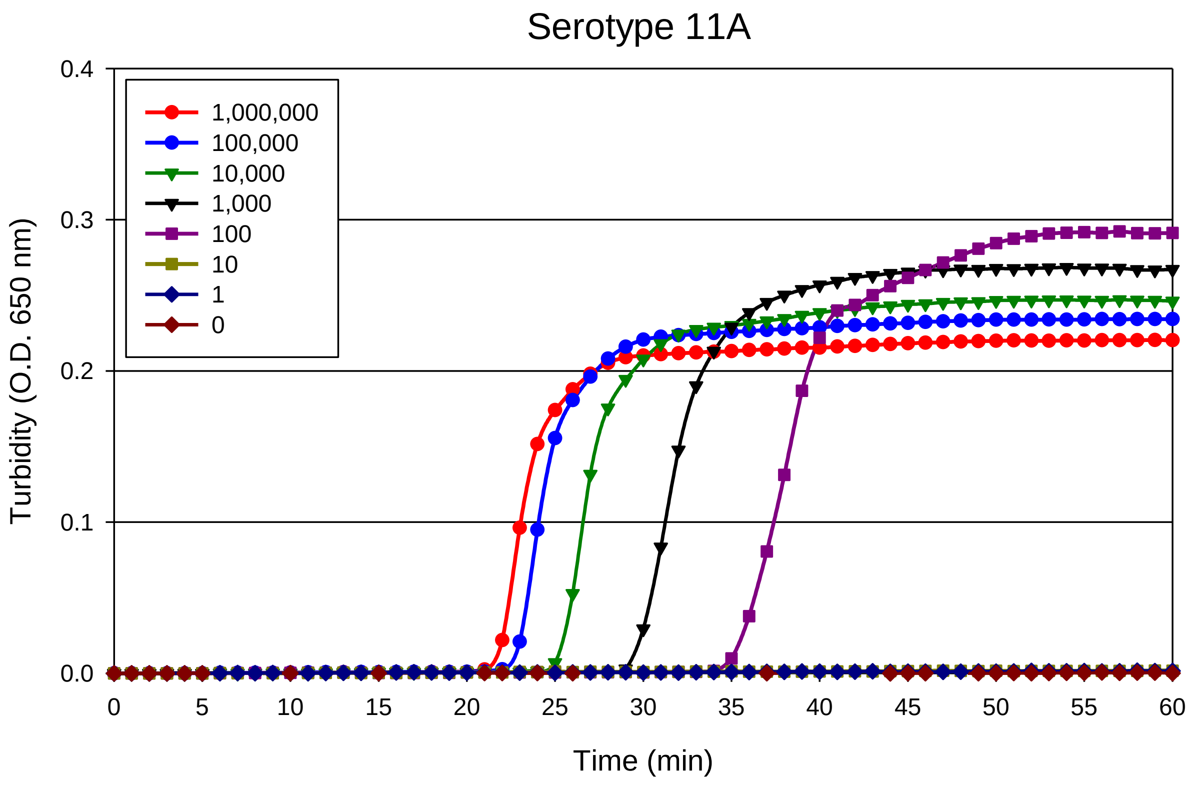

Serotype 11A
Turbidity (O.D. 650 nm)
Time (min)
0.4
0.3
0.2
0.1
0.0
0
5
10
15
20
25
30
35
40
45
50
55
60
1,000,000
100,000
10,000
1,000
100
10
1
0

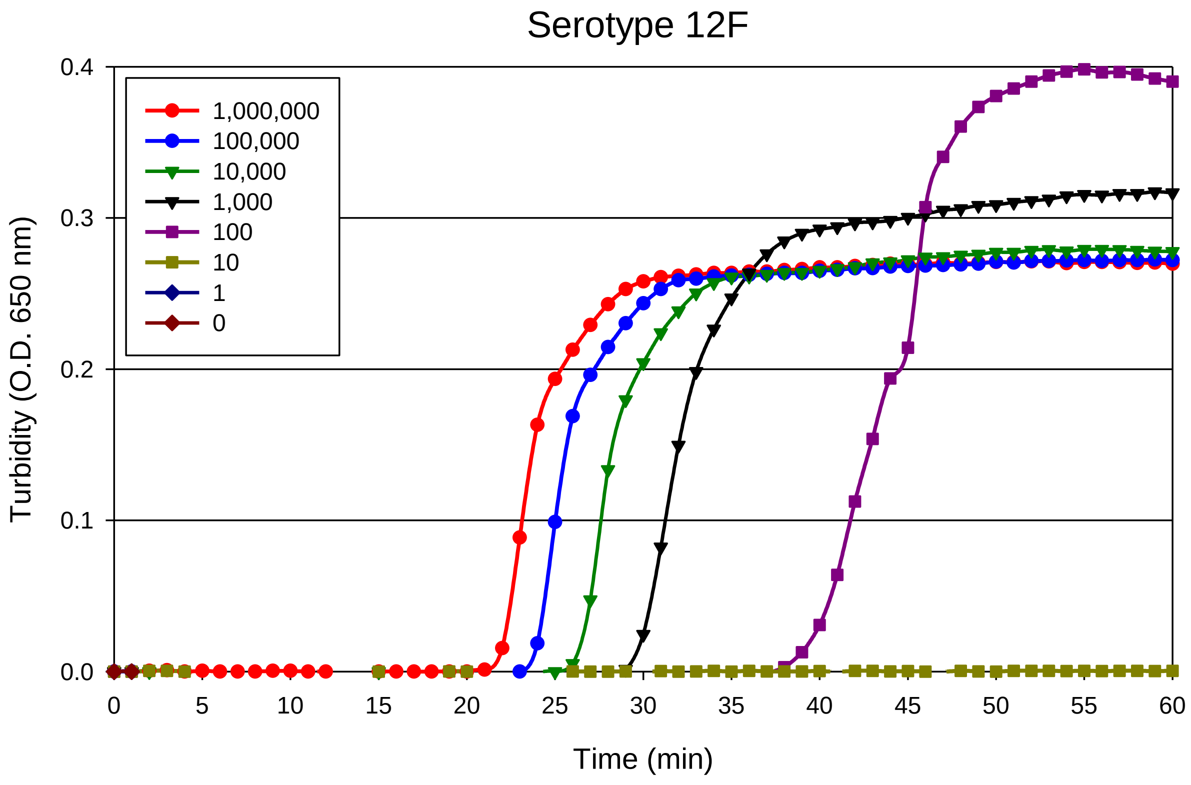

Serotype 12F
Turbidity (O.D. 650 nm)
Time (min)
0.4
0.3
0.2
0.1
0.0
0
5
10
15
20
25
30
35
40
45
50
55
60
1,000,000
100,000
10,000
1,000
100
10
1
0

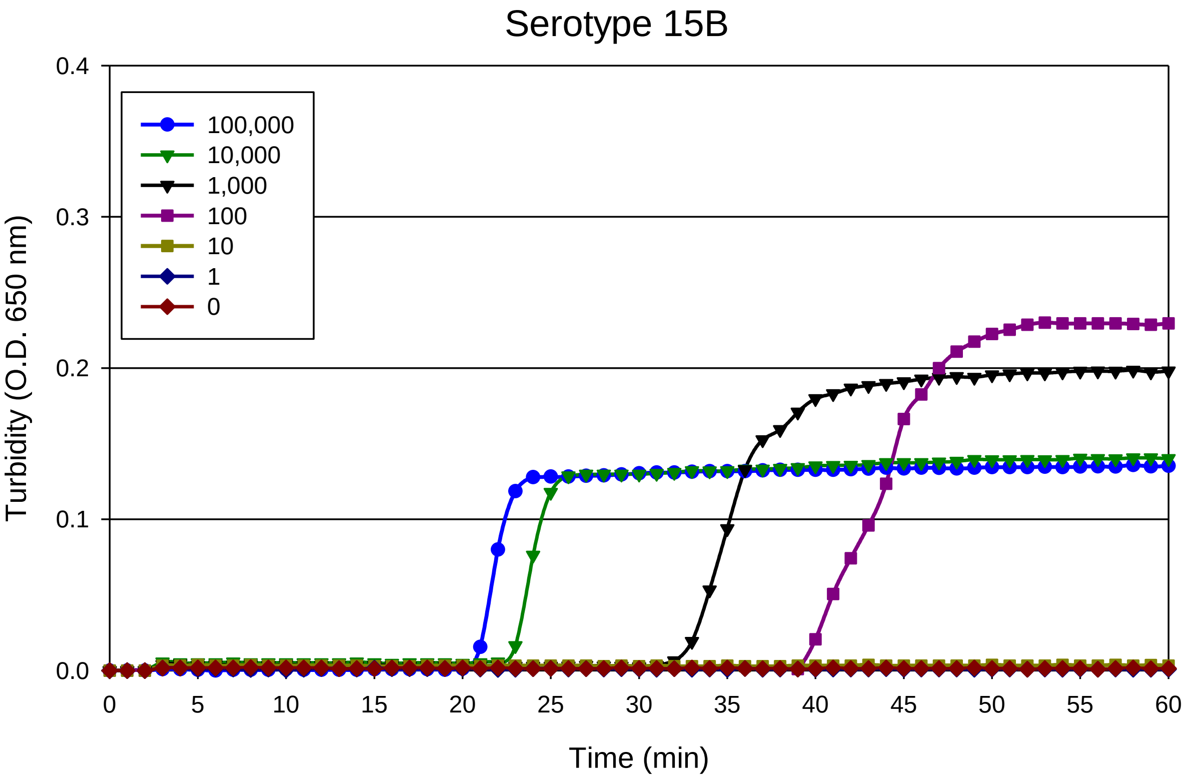
Serotype 15B
Turbidity (O.D. 650 nm)
Time (min)
100,000
10,000
1,000
100
10
1
0
0.0
0.1
0.2
0.3
0.4
0
5
10
15
20
25
30
35
40
45
50
55
60

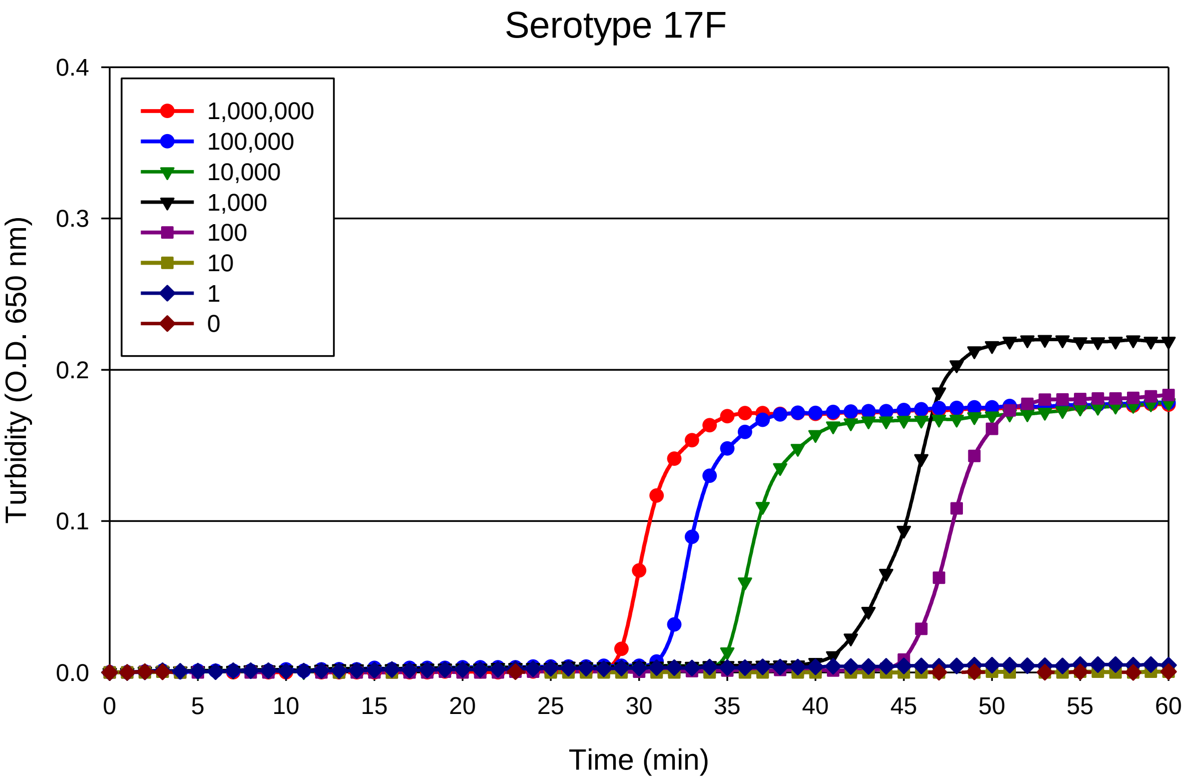
Serotype 17F
Turbidity (O.D. 650 nm)
Time (min)
1,000,000
100,000
10,000
1,000
100
10
1
0
0.0
0.1
0.2
0.3
0.4
0
5
10
15
20
25
30
35
40
45
50
55
60

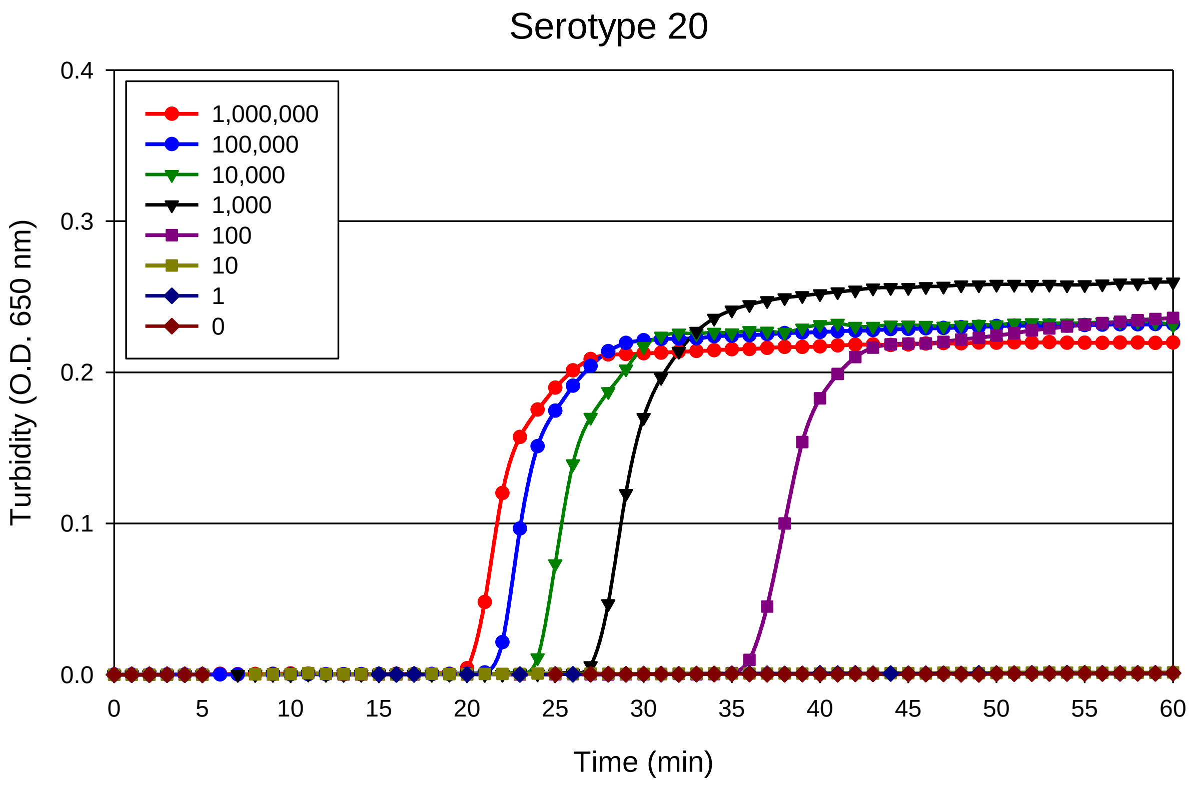
Serotype 20
Turbidity (O.D. 650 nm)
Time (min)
1,000,000
100,000
10,000
1,000
100
10
1
0
0.0
0.1
0.2
0.3
0.4
0
5
10
15
20
25
30
35
40
45
50
55
60

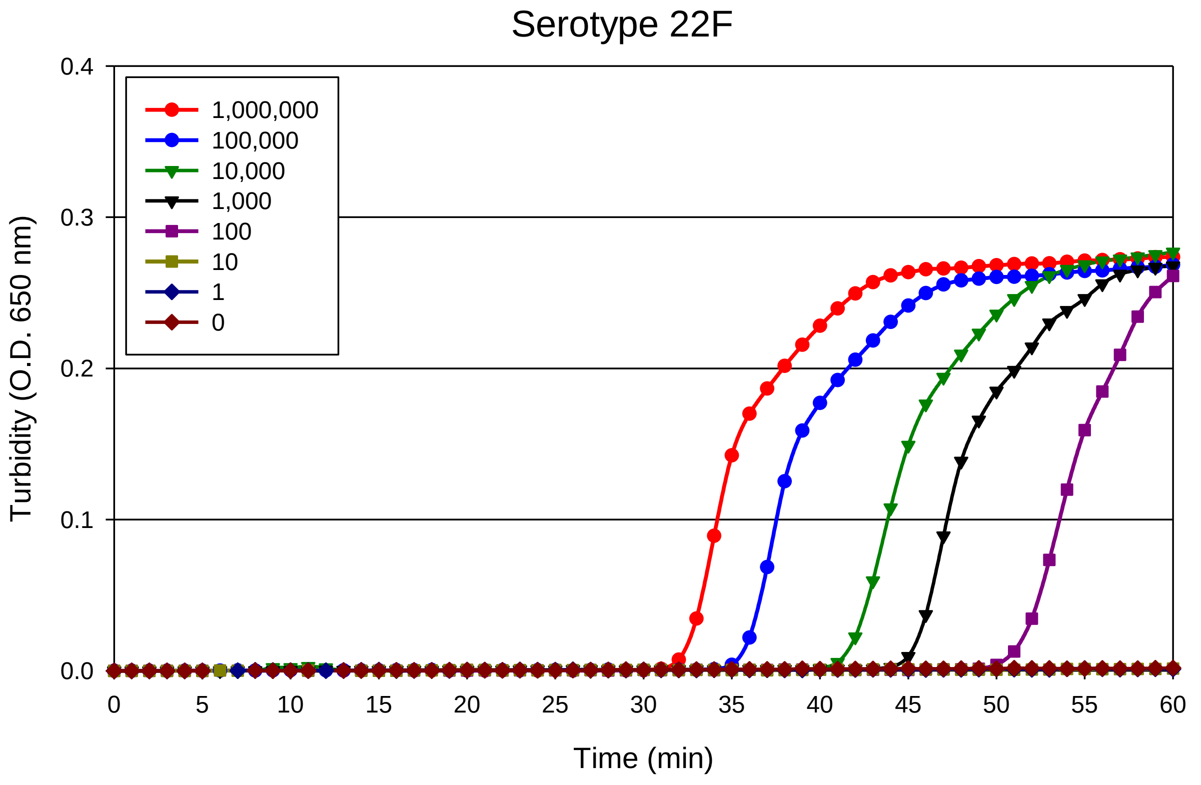
Serotype 22F
Turbidity (O.D. 650 nm)
Time (min)
1,000,000
100,000
10,000
1,000
100
10
1
0
0.0
0.1
0.2
0.3
0.4
0
5
10
15
20
25
30
35
40
45
50
55
60

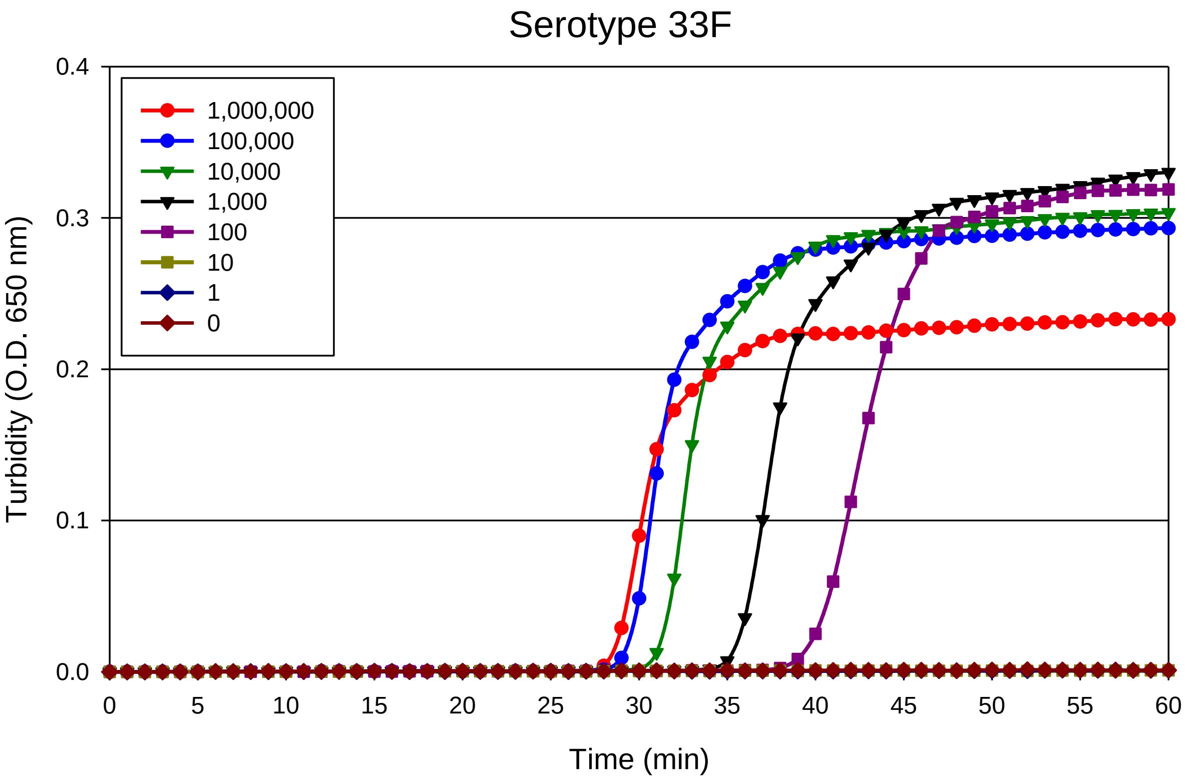
Serotype 33F
Turbidity (O.D. 650 nm)
0.0
0.1
0.2
0.3
0.4
Time (min)
0
5
10
15
20
25
30
35
40
45
50
55
60
1,000,000
100,000
10,000
1,000
100
10
1
0
